# PRL1 Promotes Glioblastoma Invasion and Tumorigenesis *via* Activating USP36-Mediated Snail2 Deubiquitination

**DOI:** 10.3389/fonc.2021.795633

**Published:** 2022-01-17

**Authors:** Wenjin Qiu, Xiaomin Cai, Kaya Xu, Shibin Song, Zumu Xiao, Yunan Hou, Xiaolan Qi, Feng Liu, Yimin Chen, Hua Yang, Liangzhao Chu, Jian Liu

**Affiliations:** ^1^ Department of Neurosurgery, The Affiliated Hospital of Guizhou Medical University, Guiyang, China; ^2^ School of Basic Medical Sciences, Guizhou Medical University, Guiyang, China; ^3^ Department of Neurosurgery, School of Medicine, Xinhua Hospital, Shanghai Jiaotong University, Shanghai, China; ^4^ Key Laboratory of Endemic and Ethnic Diseases, Ministry of Education and Key Laboratory of Medical Molecular Biology of Guizhou Province, Guizhou Medical University, Guiyang, China; ^5^ Department of Neurosurgery, The First Affiliated Hospital of Guangdong Pharmaceutical University, Guangzhou, China; ^6^ Department of Neurosurgery, Guizhou Provincial People’s Hospital, Guiyang, China

**Keywords:** glioblastoma, PRL1, epithelial mesenchymal transition, Snail2, deubiquitination

## Abstract

Regenerating liver phosphatase 1 (PRL1) is an established oncogene in various cancers, although its biological function and the underlying mechanisms in glioblastoma multiforme (GBM) remain unclear. Here, we showed that PRL1 was significantly upregulated in glioma tissues and cell lines, and positively correlated with the tumor grade. Consistently, ectopic expression of PRL1 in glioma cell lines significantly enhanced their tumorigenicity and invasion both *in vitro* and *in vivo* by promoting epithelial-mesenchymal transition (EMT). Conversely, knocking down PRL1 blocked EMT in GBM cells, and inhibited their invasion, migration and tumorigenic growth. Additionally, PRL1 also stabilized Snail2 through its deubiquitination by activating USP36, thus revealing Snail2 as a crucial mediator of the oncogenic effects of PRL1 in GBM pathogenesis. Finally, PRL1 protein levels were positively correlated with that of Snail2 and predicted poor outcome of GBMs. Collectively, our data support that PRL1 promotes GBM progression by activating USP36-mediated Snail2 deubiquitination. This novel PRL1/USP36/Snail2 axis may be a promising therapeutic target for glioblastoma.

## Introduction

High grade gliomas, especially glioblastoma multiforme (GBM), are common and highly malignant intracranial primary tumors in adults (1, 2). Although surgical resection, postoperative adjuvant chemotherapy and radiation therapy have achieved some encouraging results, the prognosis of GBM patients remains dismal with a median overall survival (OS) of approximately 12-15 months after diagnosis and a 5-year survival rate of less than 9.8% (3, 4). The high invasiveness of the GBM cells is one of the main reasons of the poor therapeutic efficacy (5). Thus, dissecting the molecular mechanisms that drive GBM invasion and tumorigenesis hold great promise in identifying novel therapeutic targets.

Phosphatases of regenerating liver (PRLs) are a subfamily of protein tyrosine phosphatases (PTPs) that encompass PRL-1, PRL-2 and PRL-3 (6), and are involved in cancer development and metastasis (7, 8). PRL1 is encoded by the human *PTP4A1* gene, and was initially identified as a highly overexpressed protein during liver regeneration after partial hepatectomy (9). PRL1 regulates cell motility, invasion, growth and survival, and neural crest specification through various pathways (10–12), and is associated with tumor development and metastasis, along with poor patient prognosis (13–15). PRL1 is aberrantly overexpressed in hepatocellular carcinoma, lung cancer, colorectal cancer and ovarian carcinoma (16–19). PRL1 mRNA is upregulated in esophageal squamous cell carcinoma (ESCC) cells compared to normal esophageal cells, and is a predictive marker of metastasis (20). In addition, PRL1 overexpression significantly promoted the invasion and migration of hepatocellular carcinoma cells by inhibiting E-cadherin expression *via* the PI3K/AKT/GSK3β pathway (18), and maintained the malignant phenotype of human lung cancer cells by activating Src (17). Studies have also shown that PRL1 plays a vital role in cell proliferation and invasion by upregulating the ERK1/2 and RhoA pathways (9). These findings suggest that PRL1 is an oncogene that promotes cancer cell invasion and migration, although its role in GBM is largely unknown.

Epithelial-mesenchymal transformation (EMT) plays a critical role in differentiation, tissue remodeling, and cancer metastasis (21, 22). It is characterized by the loss of epithelial markers (e.g. E-cadherin) and the concomitant upregulation of mesenchymal markers (such as N-cadherin, fibronectin and Vimentin) (23). EMT leads to cytoskeletal remodeling and breakdown of extracellular matrix, which allows the cells to enter the bloodstream and metastasize to distant organs. In addition, EMT is also a crucial factor for the chemoresistance of gliomas (24). EMT is driven by the Snail family of zinc-finger transcription factors, including Snail1 and Snail2 (also known as Slug) (25). Snail proteins are polyubiquitinated and degraded by the 26S proteasome, which is dependent on their phosphorylation state (26, 27). Ubiquitination plays a vital role in the post-translational modification of cellular proteins in physiological and pathological conditions including cancer (28). Deubiquitination, a reverse process mediated by deubiquitination enzymes (DUBs), blocks proteosomal degradation (29, 30) of oncoproteins and tumor suppressor proteins, and is dysregulated in cancer cells (29). USP36 is an ubiquitin specific protease (USP) that has been reportedly overexpressed in a number of human cancers, including breast and lung cancers (31). Moreover, USP36 overexpression confers therapeutic resistance in ovarian cancer tissues, and correlated with poor prognosis (32). USP36 harbors oncogenic properties and its higher expression in neuroblastoma patients correlates with poor prognosis while its downregulation significantly reduces tumor growth in neuroblastoma cell lines and xenograft models (33).

In this study, we explored potential role of PRL1 in GBM tumorigenesis and invasion, and found that PRL1 is upregulated in GBM and correlates with patient prognosis. In addition, ectopic expression of PRL1 in the GBM cell lines promoted their invasion and tumorigenesis, whereas PRL1 knockdown had the opposite effects. Mechanistically, PRL1 promoted the deubiquitination and stabilization of the EMT-inducing transcriptional factor Snail2 by activating USP36. Thus, PRL1/USP36/Snail2 axis may be a novel potential therapeutic target for GBMs.

## Materials and Methods

### Cell Culture and Reagents

The cell lines U87MG, U251, LN229, T98G, HS683 and SW1783 were purchased from the American Type Culture Collection (ATCC). Human embryonic kidney 293T (HEK293T) cells were obtained from Bena Culture Collection Technology (China). All cell lines were cultured in DMEM supplemented with 10% FBS. Normal human astrocytes (NHAs) were purchased from Lonza (Walkersville, MD) and maintained as per the manufacturer’s instructions. Cycloheximide (CHX) and puromycin were acquired from Sigma Aldrich. MG132 was purchased from Millipore, and Lipofectamine 3000 was obtained from Invitrogen.

### Patient Tumors

Sixty-two glioma samples (8 grade I, 11 grade II, 17 grade III and 26 grade IV) and 10 normal brain tissues (NBTs) were obtained from the Department of Neurosurgery, the Affiliated Hospital of Guizhou Medical University. The NBTs were obtained from patients who underwent craniotomy and decompression for traumatic brain injury. The specimens were histologically identified according to the WHO criteria by two independent neuro-pathologists. The clinicopathological characteristics of the patients are summarized in **Supplementary Tables S1, S2**. This study was approved by the Ethics Committee of Guizhou Medical University, and written informed consent was obtained from all patients.

### RNA Extraction and qRT-PCR Analysis

Total RNA was isolated using TRIzol reagent (Invitrogen) according to the manufacturer’s protocol and the first strand cDNA was synthesized with PrimeScript RT Master Mix (TaKaRa). Real-time quantitative PCR was performed according to manufacturer’s instructions using SYBR Green (Applied Biosystems). Relative expression levels of PRL1 and Snail2 mRNAs were normalized to that of GAPDH and calculated by the standard 2^-ΔΔCt^ method. The primer sequences were as follows: PRL1 forward 5’-CCAGCTCCTGTGGAAGTCAC-3’ & reverse 5’-CCATCATCAAAAGGCCAATC-3’; Snail2 forward 5’-CGAACTGGACACACATACAGTG-3’ & reverse 5’-CTGAGGATCTCTGGTTGTGGT-3’; USP36 forward 5’-AGCACTTTTCCCCCAGAACTG-3’ & reverse 5’-GGCTCCCAGATCTGCTGCTA-3’; GAPDH forward 5’-TGCACCACCAACTGCTTAGC-3’ & reverse 5’-GGCATGGACTGTGGTCATGAG-3’.

### Transfection

Human PRL1-shRNA#1 (5′-AAGCAACTTATGACACTACTC-3′), PRL1-shRNA#2 (5′- AACAGCAAGCAACTTCTGTAT-3′), USP36 shRNA#1 (5′- CGTCCGTATATGTCCCAGAAT-3′) and USP36 shRNA#2 (5′- GGAAGAGTCTCCAAGGAAA-3′) sequences were cloned into lentiviral vectors (with a 9-nt spacer followed by reverse complimentary sequence). Lentiviral constructs expressing Flag-tagged USP36, Flag-tagged USP36^C131A^ and His-tagged Snail2 were generated by cloning the respective ORFs with the N-terminal Flag or His Tag into the pCDH-CMV-MCS-EF1α-Puro vector. Plasmids coding for HA-tagged ubiquitin-Lys63 (pRK5-HA-ubiquitin-Lys63) and HA-tagged ubiquitin-Lys48 (pRK5-HA-ubiquitin-Lys48) were obtained from Addgene. Site-directed mutagenesis in USP36 was performed using the QuikChange Mutagenesis Kit (Agilent Technologies), according to the manufacturer’s instructions. The authenticity of all constructs was confirmed by DNA sequencing.

### Transwell Invasion Assay

The 24-well BD Matrigel invasion chamber (BD Biosciences) was used to evaluate cell invasion *in vitro* according to the manufacturer’s instructions. The cells were seeded in the upper chambers at the density of 2×10^4^ cells per well in serum-free medium, and the lower chambers were filled in DMEM supplemented with 10% FBS to stimulate invasion. After incubating for 24h, the cells remaining on the top of the insert membranes were swabbed, and those at the bottom well were fixed with 4% paraformaldehyde and stained with 0.1% crystal violet. All assays were performed in sextuplicate and the cells were counted in three non-overlapping fields per well under 20× magnification.

### Wound Healing Assay

The suitably transfected cells were seeded in a 6-well plate at the density of 3×10^5^ cells/well and cultured until they reach complete confluency. The monolayer was scratched with a 20 μL plastic pipette to create an artificial wound. The wound region was imaged at 0 and 24h after injury under an inverted microscope at 10x magnification, and the cell coverage area was demarcated and annotated manually.

### Colony Formation Assay

The cells were seeded into a six-well plate at the density of 5 ×10^2^ cells/well and cultured for 14 days. The colonies were washed three times with PBS, fixed with 100% methanol for 20 min, and stained with 1% crystal violet in 20% methanol for 15 min. After washing with PBS, the number of colonies was counted and the averages were calculated.

### Western Blotting

Western blotting was performed as previously described (34). The tissue homogenates or cell lysates were probed with antibodies against PRL1 (sc-130354, Santa Cruz), N-cadherin (#13116, Cell Signaling Technology), Vimentin (ab8978, Abcam), E-cadherin (#14472, Cell Signaling Technology), Twist1 (ab175430, Abcam), Twist2 (ab66031, Abcam), ZEB1 (ab203829, Abcam), β-catenin (ab16051, Abcam), β-actin (ab8227, Abcam), Snail1 (13099-1-AP, Proteintech), Snail2 (sc-166476, Santa Cruz), USP36 (NB100-40832, Novus), FLAG-tag (ab205606, Abcam), HA-tag (ab9110, Abcam) and His-tag (ab9108, Abcam).

### siRNA Library Screening

The Dharmacon siGENOME RTF SMARTpool siRNA library was used to screen for human deubiquitylases. Briefly, HEK293T cells were added to the rehydrated Dharmacon RTF siRNA library plates, and lysed 48 hours later. Endogenous Snail2 levels were detected by immunoblotting.

### Immunoprecipitation

Cells were lysed using NETN buffer containing protease inhibitors (P8340, Sigma-Aldrich), and the lysates were cleared with protein A/G beads and immunoprecipitated overnight with the indicated primary antibody at 4°C. The beads were washed three times with NETN buffer, denatured in SDS loading buffer, and the immunoprecipitated complexes were analyzed by Western blotting.

### Deubiquitination Assays

Deubiquitination assays were performed as previously described (35). HA-ubiquitinated His-Snail2 or endogenous Snail2 was immunoprecipited using the indicated antibodies in denaturing conditions. Then the ubiquitination level of Snail2 was detected using antibody against HA.

### *In Vivo* Xenografts

To induce subcutaneous xenografts, 1×10^6^ HS683 cells were inoculated subcutaneously into 5-6 weeks old male nude mice. Tumor volume was measured as 0.5×length×width^2^. To establish the orthotopic model, 5×10^5^ U87MG cells stably transduced with luciferase-expressing lentivirus were intracranially injected into 5-6 weeks old male nude mice. Tumor growth was monitored using *in vivo* bioluminescence imaging. The mice were euthanized when they appeared moribund, and the brains were perfused with 4% paraformaldehyde and embedded in paraffin. All animal experiments were conducted as per the guidelines of the Animal Welfare Ethical Review Committee of Guizhou Medical University.

### Immunohistochemistry (IHC)

The *in-situ* expression of PRL1, USP36 and Snail2 in human glioma tissues and mouse xenografts were detected by IHC as previously described (36).

### Statistical Analysis

Data was expressed as mean ± standard error of the mean. One-way analysis of variance (ANOVA) was used to compare multiple groups and two tailed student’s *t*-tests was used for two-group comparisons. The relationship between PRL1 levels and Snail2 expression was analyzed by Pearson’s correlation analysis. Survival of patients and tumor-bearing mice were analyzed by the Kaplan–Meier method by GraphPad Prism 7 software. SPSS 24.0 was used for all statistical analyses. *p* < 0.05 was considered statistically significant.

## Results

### PRL1 Is Overexpressed in GBM Tissues and Cell Lines

**Figure 1:**
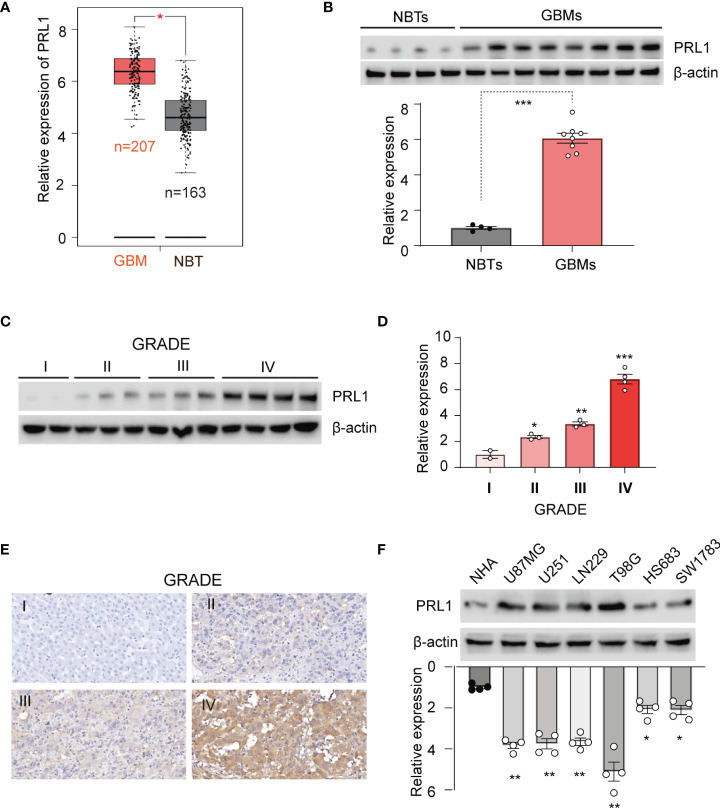
PRL1 is overexpressed in GBM tissues and cell lines. **(A)** PRL1 expression levels analyzed using GEPIA database. **(B)** Immunoblot showing PRL1 protein levels in randomly selected 4 NBTs and 8 GBM samples. Lower panel, quantification of immunoblot results. **(C)** Immunoblot showing PRL1 protein levels in glioma tissues of different clinical grades. **(D)** Quantification results of **(C)**. **(E)** Representative images of glioma tissues (grade I-IV) showing *in situ* PRL1 protein expression. **(F)** Immunoblot showing PRL1 protein levels in NHA and six glioma cell lines. The lower panel is quantification results. *p < 0.05; **p < 0.01; ***p < 0.001.

PRL1 expression levels were first analyzed in 163 GBM tissues and 207 NBTs using GEPIA (http://gepia.cancer-pku.cn/). As shown in **Figure 1A**, PRL1 was significantly upregulated in the GBM specimens compared to NBTs. To determine whether PRL1 plays a role in the invasiveness and progression of GBM, we analyzed PRL1 protein levels in 10 NBTs and 26 grade IV GBM samples using Western blot, and detected markedly high levels in the tumors in a subset of NBT/GBM tissues (**Figure 1B**). In a cohort of 62 glioma specimens, PRL1 protein expression levels were strongly correlated with the clinical grading (**Figures 1C–E**). Consistent with these findings, PRL1 protein was also found significantly upregulated in multiple GBM cell lines (U87MG, U251, LN229 and T98G) compared to the normal human astrocytes (NHAs) and grade III glioma cell lines (HS683 and SW1783) (**Figure 1F**). Taken together, these data suggest that PRL1 likely plays an oncogenic role in GBM.

### PRL1 Increased Glioma Cell Invasion, Migration and Tumor Formation by Promoting EMT

**Figure 2:**
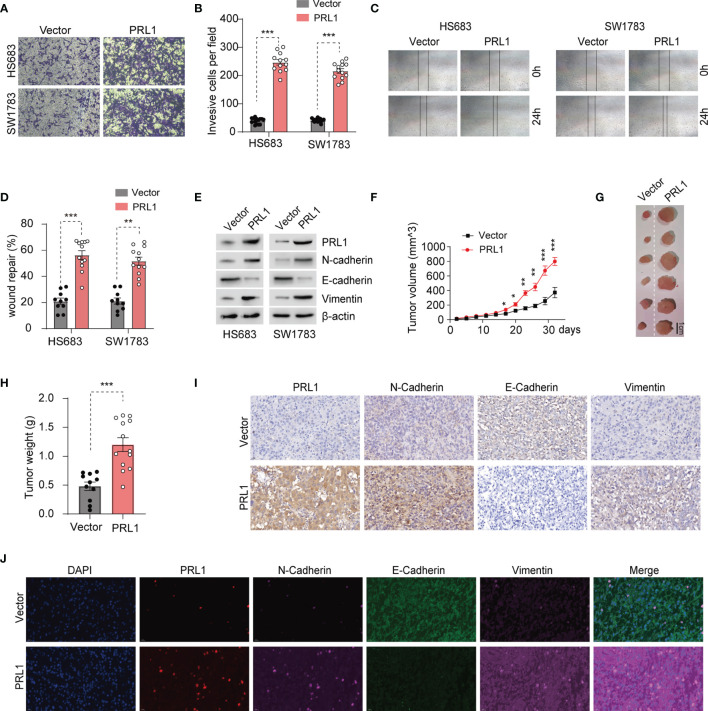
PRL1 promotes glioma cell invasion, migration and tumor formation by raising EMT. **(A)** Representative images of transwell invasion assay of HS683 and SW1783 cells transduced with PRL1 or empty vector. **(B)** Number of invading cells in the indicated groups. **(C)** Representative images showing *in vitro* wound coverage by HS683 and SW1783 cells transduced with PRL1 or empty vector. **(D)** Wound coverage area in the indicated groups. **(E)** Immunoblot showing the levels of EMT-associated proteins in HS683 and SW1783 cells transfected with PRL1 or empty vector. **(F)** Growth curve of subcutaneous tumor xenografts derived from HS683 cells stably transduced with PRL1 or empty vector. *n* =13 each group. **(G)** Representative images of subcutaneous xenograft tumor. **(H)** Xenografted tumor weight in the indicated groups. *n* =13 each group. **(I)** Representative images of subcutaneous xenograft tissues showing *in situ* expression of PRL1, N-cadherin, Vimentin and E-cadherin. **(J)** Representative immunofluorescence images of subcutaneous xenograft tissues showing *in situ* expression and colocalized signals of PRL1, N-cadherin, Vimentin and E-cadherin. *p < 0.05; **p < 0.01; ***p < 0.001.

PRL1 plays a vital role in the invasion and metastasis of various tumors (18). To determine whether PRL1 overexpression in glioma is correlated to their invasiveness and migration ability, we were successful in ectopically expressing this protein in HS683 and SW1783 cells (**Supplementary Figure S1A**). We found that overexpression of PRL1 significantly enhanced their invasion through Matrigel (**Figures 2A, B**), and increased *in vitro* migration and wound coverage (**Figures 2C, D**). EMT is a known driver of cancer cell invasion and migration, and is characterized by the upregulation of epithelial markers and downregulation of mesenchymal proteins (37). We found that overexpression of PRL1 significantly increased N-cadherin and Vimentin levels, and decreased the level of E-cadherin (**Figure 2E** and **Supplementary Figure S1B**). Furthermore, the HS683 cells overexpressing PRL1 significantly increased the number of colonies compared to the control cells (**Supplementary Figure S1C**). Consistent with these *in vitro* findings, PRL1-overexpressing HS683 cells grew more rapidly in nude mice and formed significantly larger tumors compared to the control cells (**Figures 2F** **–H**). Furthermore, PRL1 overexpression increased the levels of N-cadherin and Vimentin, and decreased that of E-cadherin in the tumor sections (**Figures 2I, J**). Taken together, these data indicate that PRL1 may promote the invasion, migration and tumorigenesis of glioma cells by accelerating EMT.

### PRL1 Knockdown in GBM Cells Blocked EMT and Inhibited the Malignant Phenotype

**Figure 3:**
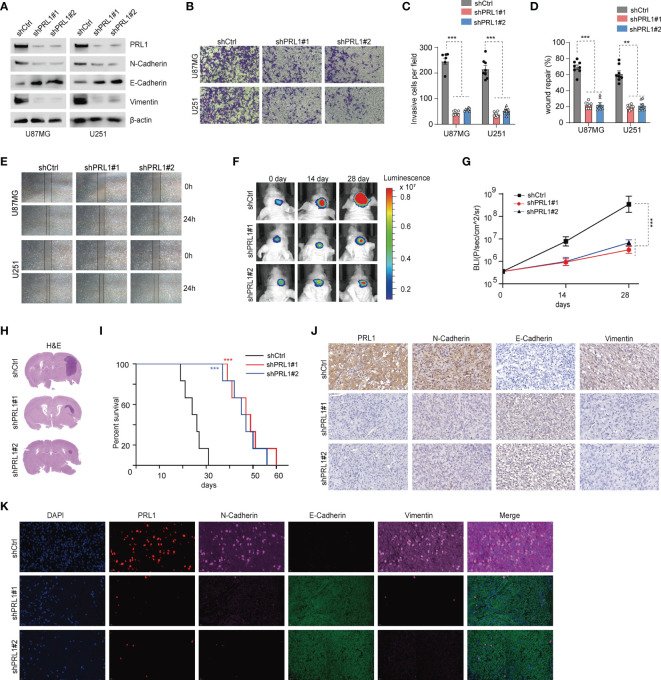
Knockdown of PRL1 blocks EMT in GBM cells and inhibits the malignant potential. **(A)** Immunoblot showing PRL1 EMT-associated protein levels in U87MG and U251 cells transduced with two small hairpin interference RNA constructs (shPRL1#1, shPRL1#2) or scrambled control vector (shCtrl). **(B)** Representative images of transwell invasion assay of U87MG and U251 cells transduced with shPRL1#1, shPRL1#2 or shCtrl. **(C)** Quantification of the numbers of invading cells in the indicated groups. **(D)** Wound coverage area in the indicated groups. **(E)** Representative photomicrograph of wound coverage area in the indicated groups in two cell lines. **(F)** Representative *in vivo* bioluminescent images of intracranial GBM xenografts derived from U87MG cells transduced with shPRL1#1, shPRL1#2 or shCtrl. Colored scale bars represent photons/s/cm^2^/steradian. *n* = 6 each group. **(G)** Bioluminescence was quantified in tumors from three groups. **(H)** Representative images of HE-stained intracranial xenografts. **(I)** Kaplan–Meier survival curves of tumor-bearing mice in the indicated groups. **(J)** Representative images of subcutaneous xenograft tissues showing *in-situ* expression of PRL1, N-cadherin, Vimentin and E-cadherin. **(K)** Representative fluorescent images of subcutaneous xenograft tissues showing *in situ* expression and colocalization of PRL1, N-cadherin, Vimentin and E-cadherin. **p < 0.01; ***p < 0.001.

To further explore the mechanistic role of PRL1 in regulating GBM cell invasion and migration, we knocked down PRL1 in U87MG and U251 cells using multiple small hairpin interference RNA sequences (shRNAs, **Figure 3A**). PRL1 silencing significantly decreased N-cadherin and Vimentin levels, and increased that of E-cadherin (**Figure 3A** and **Supplementary Figure S2A**). The transwell-Matrigel invasion assay (**Figures 3B, C**) and wound healing assay (**Figures 3D, E**) further confirmed that PRL1 knockdown significantly reduced the invasiveness and migration capacity of the glioma cells respectively. Knocking down PRL1 also markedly reduced the number of colonies formed by the U87MG cells (**Supplementary Figure S2B**). To further assess the effect of PRL1 downregulation *in vivo*, luciferase-expressing U87MG cells were transfected with a lentiviral vector containing shRNA against PRL1 (shPRL1) or scrambled control sequence (shCtrl) and intracranially injected into nude mice. Compared to the control group, knocking down PRL1 significantly inhibited tumor growth (**Figures 3F** **–H**) and prolonged the survival of tumor-bearing mice (**Figure 3I**). Furthermore, *in-situ* N-cadherin and Vimentin expression levels were markedly lower, and E-cadherin was upregulated in the PRL1-knockdown xenografts compared to the control (**Figures 3J, K**). These results strongly suggest that loss of PRL1 may impair EMT in glioma cells.

### PRL1 Knockdown Increased Snail2 Polyubiquitination and Proteasomal Degradation

**Figure 4:**
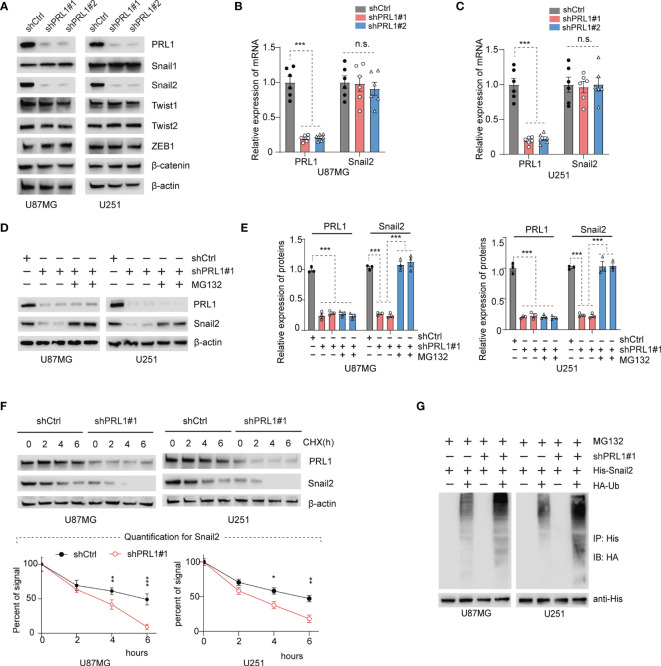
PRL1 depletion increases Snail2 polyubiquitination and proteasomal degradation. **(A)** Representative immunoblot showing PRL1, Snail1, Snail2, Twist1, Twist2, ZEB1 and β-catenin levels in U87MG and U251 cells transduced with shPRL1#1, shPRL1#2 or shCtrl. **(B, C)** Quantification of PRL1 and Snail2 mRNA levels in U87MG **(B)** and U251 **(C)** cells transduced with shPRL1#1, shPRL1#2 or shCtrl. **(D)** Immunoblot showing PRL1 and Snail2 levels in U87MG and U251 cells transduced with shPRL1#1 or shCtrl in the presence or absence of MG132 (20 μM). **(E)** Quantification of results in **(D). (F)** Half-life of Snail2 protein in U87MG and U251 transduced with shPRL1#1 or shCtrl and treated with cycloheximide (100 µg/mL) for varying durations. Quantifications of Snail2 expression normalized to β-actin are plotted, with two-way ANOVA and Sidak’s multiple comparison *post hoc* tests. **(G)** Immunoblot showing ubiquitin-conjugated His-Snail2 pulled down from control or PRL1-silenced U87MG and U251 cells co-transfected with HA-tagged ubiquitin and His-tagged Snail2. *p < 0.05; **p < 0.01; ***p < 0.001; *n.s.*, not significant.

EMT is regulated at the molecular level by several transcription factors such as Snail1, Snail2, Twist1, Twist2, ZEB1 and β-catenin (38). As shown in **Figure 4A** and **Supplementary Figure S3A**, we found that only Snail2 levels were markedly decreased in the PRL1-kncokdown U87MG and U251 cells lines, whereas the other EMT-related transcription factors were unaffected. As such, we hypothesized that PRL1 regulates EMT in the GBM cells by activating Snail2. However, no significant differences were seen in the levels of Snail2 mRNA (**Figures 4B, C**) between the control and PRL1-kncokdown cells in both U87MG and U251 lines, which suggests that PRL1 does not likely regulate Snail2 at the transcriptional level. We next treated PRL1-knockdown or shCtrl U87MG and U251 cells with the proteasome inhibitor MG132, and found that the latter restored the levels of Snail2 protein in cells lacking PRL1 (**Figures 4D**, **E** - quantifications).

To further ascertain whether PRL1 affects the stability of Snail2 protein, we treated the control and PRL1-knockdown U87MG/U251 cells with the protein synthesis inhibitor cycloheximide (CHX, 100µg/ml). As shown in **Figure 4F** and **Supplementary Figure S3B**, the half-life of Snail2 was significantly shortened in cells lacking PRL1, indicating that loss of PRL1 may accelerate the degradation of Snail2. Indeed, the combination of PRL1 knockdown and CHX treatment rapidly decreased Snail2 protein levels (**Figure 4F**). It has been previously shown that the ubiquitin proteasome system (UPS) controls the degradation and turnover of multiple target proteins including those of the Snail family (1, 39). To determine whether PRL1 mediates Snail2 proteolysis *via* the UPS pathway, we co-transfected the PRL1-knockdown or control GBM cells with plasmids encoding HA-tagged ubiquitin and His-tagged Snail2, and treated them with 20 μM MG132. Silencing PRL1 in both U87MG and U251 cells significantly decreased Snail2 protein levels and enhanced its ubiquitination compared to control cells (**Figure 4G** and **Supplementary Figure S3C**). Taken together, these findings indicate that PRL1 stabilizes Snail2 in GBM cells through polyubiquitination and targeted proteasome degradation.

### PRL1 Stabilizes Snail2 by Activating USP36

**Figure 5:**
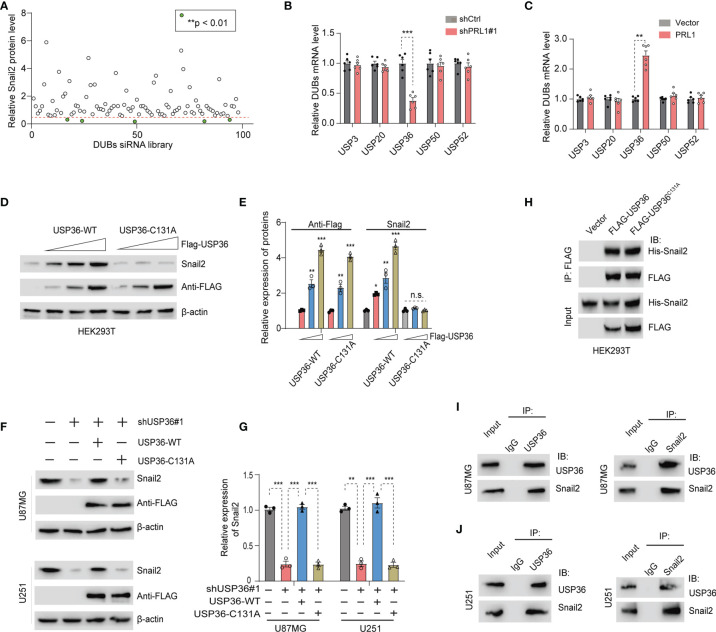
PRL1 stabilizes Snail2 by activating USP36. **(A)** Quantification of immunoblot results showing Snail2 protein levels in HEK293T cells transfected with siRNA targeting multiple DUB genes. **(B, C)** Quantification of USP36 mRNA levels in HEK293T cells with PRL1 knockdown **(B)** or overexpression **(C)**. **(D)** Immunoblot showing Snail2 protein levels in HEK293T cells transfected with USP36 wild-type (WT) or USP36 C131A. **(E)** Quantification of **(D)** Immunoblot showing Snail2 protein levels in U87MG and U251 cells co-transfected with Flag-USP36 WT or Flag-USP36 C131A with shUSP36. **(F)** USP36 depletion significantly decreased Snail2 protein levels in the U87MG and U251 cells, which was restored by overexpression of USP36-WT but not USP36-C131A. **(G)** Quantification of **(F)**. **(H)** Immunoblot showing Snail2-USP36 complexes precipitated by anti-Flag from HEK293T cells transfected with His-Snail2 with or without Flag-USP36 WT or Flag-USP36 C131A. **(I, J)** Reciprocal co-immunoprecipitation of endogenous USP36 and Snail2 in U87MG **(I)** and U251 **(J)** cells. *p < 0.05; **p < 0.01; ***p < 0.001, n.s., not significant.

Deubiquitinating enzymes (DUBs) are a broad group of proteases that directly remove ubiquitin groups from proteins, thereby regulating ubiquitin-dependent signaling pathways (30, 39). To identify potential DUBs involved in Snail2 protein degradation, we silenced the existing 98 DUBs in HEK293T cells using the DUB siGENOME RTF library siRNAs. Knocking down USP3, USP20, USP36, USP50 and USP52 significantly reduced Snail2 protein levels by more than 2-fold (**Figure 5A** and **Supplementary Figure S4**). However, only USP36 mRNA levels were significantly downregulated in the PRL1-knockdown cells (**Figure 5B**) and conversely upregulated in the PRL1-overexpressing cells (**Figure 5C**), whereas the other DUB mRNAs were unaltered in either conditions. Previous studies have shown that USP5, USP10 and USP20 can stabilize Snail2 expression levels in various solid tumors (26, 39, 40). In contrast, USP5 and USP10 mRNA levels were unaffected by changes in PRL1 expression (**Supplementary Figures S5A, B**). Based on these results, we hypothesized that PRL1 protects Snail2 from ubiquitin-mediated degradation by activating USP36. We transfected HEK293T cells with Flag-tagged wild-type (WT) or the catalytically-inactive C131A mutant USP36, and found that overexpression of USP36-WT, but not USP36-C131A, elevated Snail2 protein in a dose-dependent manner (**Figures 5D, E**). Furthermore, USP36 depletion significantly decreased Snail2 protein levels in the U87MG and U251 cells, which was restored by overexpression of USP36-WT but not USP36-C131A (**Figures 5F, G**). To determine whether USP36 directly interacts with Snail2, HEK293T cells were co-transfected with Flag-tagged USP36-WT or USP36-C131A along with His-tagged Snail2. Co-immunoprecipitation (Co-IP) confirmed that both wild type and mutant USP36 bound to Snail2 (**Figure 5H**), indicating that the DUB activity of USP36 was independent of its interaction with Snail2. Furthermore, Co-IP also demonstrated a direct physical interaction between endogenous USP36 and Snail2 proteins in U87MG and U251 cells (**Figures 5I, J**). Taken together, these data suggest that PRL1 stabilizes Snail2 protein in GBM cells by activating USP36.

### USP36 Stabilizes Snail2 Through Deubiquitination

**Figure 6:**
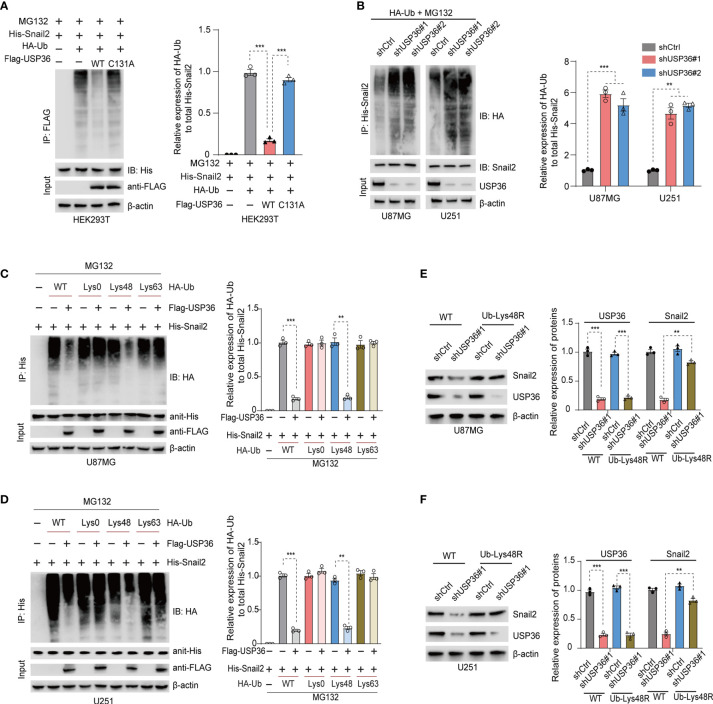
USP36 stabilizes Snail2 through deubiquitination. **(A)** Immunoblot showing Snail2 levels in HEK293T cells transfected with His-Snail2, HA-Ub and Flag-USP36-WT or Flag-USP36-C131A in the presence 20 μM MG132. Right panel, quantification results. **(B)** Immunoblot showing Snail2-Ub complexes precipitated by anti-His and probed with anit-HA, Snail2 and USP36 antibodies from GBM cells transfected with HA-Ub and shCtrl or sh-USP36. Right panel, quantification. **(C, D)** Snail2 ubiquitylation linkage in U87MG **(C)** and U251 **(D)** cells co-transfected with His-Snail2, Flag-USP36 and HA-Ub Lys0, Lys48-only, or Lys63-only plasmids. Right panel, quantification. **(E, F)** Immunoblot showing Snail2 levels in U87MG **(E)** and U251 **(F)** cells co-transfected with Ub-WT or Ub-Lys48R and shCtrl or shUSP36, with quantifications on *right* panel. **p < 0.01; ***p < 0.001.

To determine whether USP36 directly deubiquitinates Snail2, we co-transfected HEK293T cells with His-Snail2, HA-ubiquitin and Flag-USP36 (WT or C131A). IP of MG132-treated cells with the anti-Snail2 antibody indicated heavy ubiquitination of Snail2, which was near completely abolished by overexpression of USP36-WT but not USP36-C131A (**Figure 6A**). On the other hand, knocking down USP36 significantly increased Snail2 polyubiquitylation in U87MG and U251 cells (**Figure 6B**). Lys48- or Lys63-linked chains are the two major forms of polyubiquitin chains. Lys48-linked ubiquitin chains serve as the main targeting signals for the proteasome, whereas lys63-linked ubiquitin chains are involved in the endosomal/lysosomal-dependent degradation pathway (41, 42). We found that overexpression of USP36 effectively disassembled Lys48-linked polyubiquitylation of Snail2 in U87MG and U251 cells, but had no significant effect on Lys63-linked polyubiquitylation (**Figures 6C, D**). In addition, forced expression of a Lys48-resistant (Lys48R) form of ubiquitin in USP36-knockdown U87MG and U251 cells restored Snail2 levels (**Figures 6E, F**). Taken together, Lys48-linked polyubiquitination plays a vital role in USP36-mediated de-ubiquitination of Snail2.

### The Oncogenic Function of PRL1 in GBM Cells Is Mediated by Snail2

**Figure 7:**
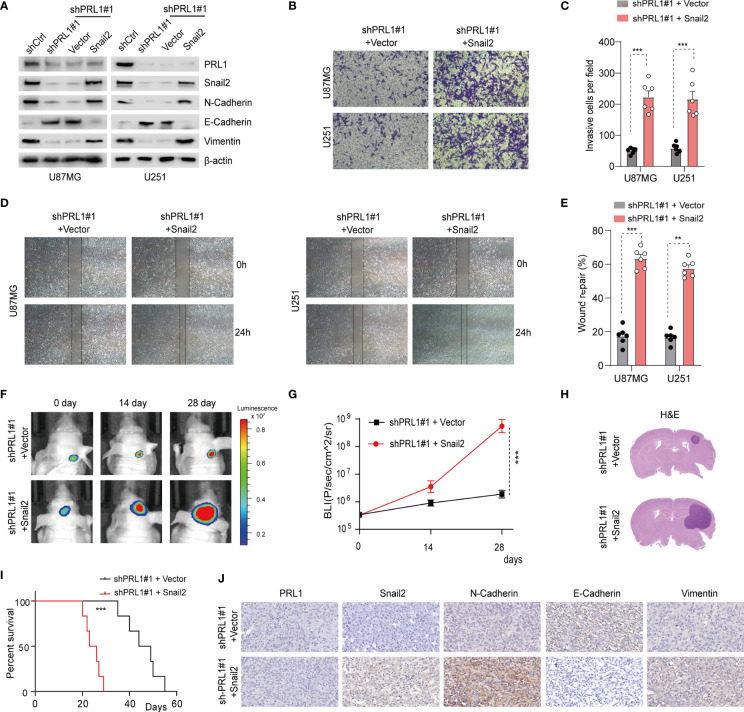
Snail2 mediates the oncogenic effects of PRL1 in GBM. **(A)** Immunoblot showing expression levels of PRL1, Snail2 and EMT-related proteins in U87MG and U251 cells co-transfected with shPRL1#1/shCtrl or Snail2/control vector. **(B)** Representative images of transwell invasion assay of U87MG and U251 cells transduced with shPRL1#1 and Snail2 or empty vector. **(C)** Number of invading cells in the indicated groups. **(D)** Representative images of wound coverage by U87MG and U251 cells transduced with shPRL1#1 and Snail2 or empty vector. **(E)** Quantification of **(D)** wound coverage area in the indicated groups. **(F)** Representative *in vivo* bioluminescent images of intracranial GBM xenografts derived from U87MG cells transduced with shPRL1#1 and Snail2 or empty vector. *n* = 6 each group. **(G)** Bioluminescence was quantified in tumors from three groups at 14 and 28 days post xenograft inoculation. **(H)** Representative images of HE-stained intracranial xenografts from the indicated groups. **(I)** Kaplan–Meier survival analysis of tumor-bearing mice in the indicated groups. **(J)** Representative images of orthotopic xenograft tissues showing *in-situ* expression of PRL1, N-cadherin, Vimentin and E-cadherin. **p < 0.01; ***p < 0.001.

To further clarify the role of Snail2 in PRL1-induced GBM progression, Snail2 was overexpressed in U87MG and U251 cells co-transfected with shPRL1. As shown in **Figure 7A** and **Supplementary Figure S6A**, Snail2 overexpression reversed changes in EMT biomarkers induced by PRL1 knockdown. Furthermore, the inhibitory effects of PRL1 silencing on *in vitro* invasion (**Figures 7B, C**) and migration (**Figures 7D, E**) of the GBM cell lines were also abrogated by the overexpression of Snail2. Moreover, the suppressive effect of PRL1 depletion on proliferation of U87MG cells could be largely rescued by Snail2 (**Supplementary Figure S6B**). Consistent with these observations, the loss of *in vivo* tumorigenicity of PRL1-knockdown cells was recovered in the presence of ectopic Snail2, as measured in terms of orthotopic xenograft growth (**Figures 7F** **–H**) and the survival of tumor-bearing mice (**Figure 7I**). Finally, N-cadherin and Vimentin proteins were upregulated, while E-cadherin was downregulated in tumors overexpressing Snail2 in the absence of endogenous PRL1 (**Figure 7J**). Taken together, these data suggest that Snail2 is a key mediator of the oncogenic effects of PRL1 in GBM.

### PRL1 Is Positively Correlated With Snail2 and Predicts Poor Outcome of GBM

**Figure 8:**
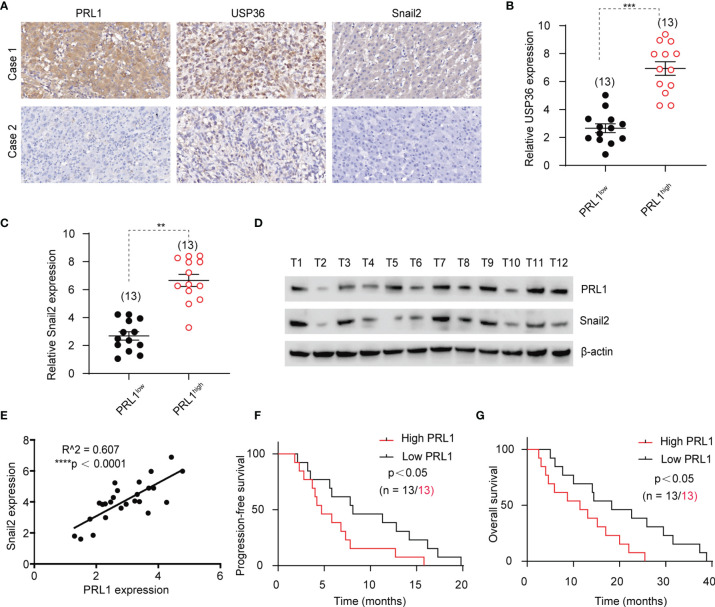
PRL1 is positively correlated with Snail2 and predicts poor outcome of GBM. **(A)** Representative images of glioma tissues showing *in situ* expression of PRL1, USP36 and Snail2 proteins in two human GBM cases. **(B, C)** Relative levels of USP36 **(B)** and Snail2 **(C)** proteins in 13 PRL1^high^ and 13 PRL1^low^ GBM specimens. **(D)** Immunoblot showing PRL1 and Snail2 levels in 12 freshly collected clinical GBM tissue samples. **(E)** Spearman correlation analysis between PRL1 and Snail2 levels in 26 GBM tissue samples. **(F, G)** Kaplan-Meier curves showing progression-free **(F)** and overall **(G)** survival of GBM patients with high (*n* = 13) or low (*n* = 13) PRL1 expression. **p < 0.01; ***p < 0.001; ****p < 0.0001.

To assess the clinical relevance of our findings, 26 glioblastoma patients’ specimens were immune-stained for PRL1, USP36 and Snail2. There was a strong positive correlation between the *in situ* expression levels of PRL1, USP36 and Snail2 in these tissues (**Figures 8A** **–C**), which was further corroborated by analyzing the total protein extracts of 26 freshly collected GBM tissue samples (**Figures 8D, E** and **Supplementary Figure S7**). In addition, Kaplan-Meier survival analysis indicated that patients with high expression of PRL1 (n = 13) had worse disease-free and overall survival compared to the PRL1^low^ group (n = 13) (**Figures 8F, G**). These results confirm the clinical relevance of the PRL1/USP36/Snail2 axis, and establish PRL1 as a potential prognostic marker for GBM patients.

## Discussion

The high invasiveness of glioma cells is one of the major factors responsible for the poor prognosis of GBM, although the underlying mechanisms governing tissue invasion and tumorigenesis are not completely understood (2, 3, 43). PRL1 plays a vital role in the invasion and dissemination of hepatocellular, gastric, ovarian, colon and lung cancers (44). However, its function in glioblastoma invasion and tumorigenesis is largely unknown.

We found that PRL1 was significantly upregulated in GBM issues and cell lines compared to the normal brain tissues and astrocytes respectively, and the overexpression of PRL1 correlated with higher tumor grade and worse prognosis. Ectopic expression of PRL1 in glioma cells significantly increased their tumorigenicity *in vitro* and *in vivo*, suggesting an oncogenic function. Specifically, gain of function of PRL1 in glioma cell lines facilitated EMT, characterized by the upregulation of N-cadherin and Vimentin, and reduction of E-cadherin levels. Conversely, knockdown of PRL1 suppressed GBM cell invasion *in vitro* and tumorigenesis *in vivo*, which indicates that PRL1 as a potential therapeutic target.

Previous studies have shown that the EMT-related transcription factor Snail2 is dysregulated in multiple cancers (40, 45). Snail2 is upregulated during EMT (46) and itself downregulates E-cadherin in lung carcinoma and hepatocellular carcinoma, which induces a mesenchymal phenotype and facilitates tumor cell metastasis (47, 48). The Snail family of proteins can be rapidly degraded by the E3 ubiquitin ligases and proteasomes (27) in response to cellular stresses such as hypoxia, chemotherapeutic drugs, oxidative stress or irradiation (49). As a phosphatase, PRL1 plays a key role in signal transduction pathways that activate downstream transcription factors by regulating the phosphorylation levels of growth factors (17). We therefore hypothesized that PRL1 is functionally coupled with and capable of modulating Snail2 function. Our findings show for the first time that the loss of function of PRL1 facilitates Snail2 polyubiquitination and proteasomal degradation, resulting in the reversal of key molecular signatures associated with PRL1-induced EMT. Thus, PRL1 stabilizes Snail2 by blocking proteolysis, which is crucial for its oncogenic activity in GBM.

Protein ubiquitination is dynamically regulated by ubiquitin ligases and deubiquitinases (50). The latter isolates ubiquitin from substrates and terminate ubiquitin-dependent signaling (51). We tested five DUBs in glioma cells that may be involved in regulating Snail2 degradation, of which only USP36 was affected by PRL1 overexpression or knockdown. USP36 regulates various cellular events by deubiquitinating target proteins such as c-myc and H2B (31, 52). We found that USP36 suppressed the polyubiquitination and proteasomal degradation of Snail2 by directly removing ubiquitin conjugates. In contrast, knocking down USP36 promoted Snail2 proteolysis *via* polyubiquitination. These results indicate that the oncogenic effects of PRL1 in GBM is mediated *via* USP36-dependent stabilization of Snail2, which is consistent with our observation that Snail2 overexpression completely reverses the impact of PRL1 knockdown.

Based on our observations, PRL1 expression may have significant value as an indicator of unfavorable progression for glioblastoma patients. We provide compelling evidence that decreased expression of PRL1 inhibits cell invasion and tumorigenesis effects on EMT mediated *via* changes in USP36-mediated Snail2 stability. Our studies indicated that a strong positive correlation between the expression levels of PRL1, USP36 and Snail2 in GBM tissues, and patients with high expression of PRL1 had worse disease-free and overall survival. These results substantiate the clinical relevance of the PRL1/USP36/Snail2 axis, and establish PRL1 as a potential prognostic marker for GBM patients.

In conclusion, our studies reveal that PRL1 promotes GBM invasion, migration and progression by activating USP36-mediated Snail2 deubiquitination. This novel PRL1/USP36/Snail2 axis plays a critical role as the underlying mechanisms of GBM progression, and may be a potential therapeutic target.

## Data Availability Statement

The original contributions presented in the study are included in the article/**Supplementary Material**. Further inquiries can be directed to the corresponding authors.

## Ethics Statement

The studies involving human participants were reviewed and approved by The Ethical Committee of Guizhou Medical University. The patients/participants provided their written informed consent to participate in this study. Written informed consent was obtained from the individual(s) for the publication of any potentially identifiable images or data included in this article. The animal study was reviewed and approved by The Animal Welfare Ethical Review Committee of Guizhou Medical University.

## Author Contributions

WQ, LC, and JL designed the experiments. WQ, XC, and KX conducted most of the experiments. SS and ZX collected the clinical tissue samples. WQ, YH, FL, and XQ analyzed the data. WQ and KX wrote the manuscript, and YC, HY, LC and JL reviewed and revised the manuscript. All authors contributed to the article and approved the submitted version.

## Funding

This study was supported by grants from the Special project of cultivating new academic seedlings of Guizhou Medical University [Grant No. QianKeHe Platform talents (2018) 5779-35], Cultivate project 2020 for National Natural Science Foundation of China, the Affiliated hospital of Guizhou Medical University (gyfynsfc (2020)-15), National Natural Science Foundation of China [Grant No. 81560409], Guizhou Provincial Natural Science Foundation [Grant No. QianKeHe (2016) support 2905], and Guizhou Provincial Health Commission Foundation [Grant No. gzwjkj2019-1-149].

## Conflict of Interest

The authors declare that the research was conducted in the absence of any commercial or financial relationships that could be construed as a potential conflict of interest.

## Publisher’s Note

All claims expressed in this article are solely those of the authors and do not necessarily represent those of their affiliated organizations, or those of the publisher, the editors and the reviewers. Any product that may be evaluated in this article, or claim that may be made by its manufacturer, is not guaranteed or endorsed by the publisher.

## Supplementary Material

Supplementary Figure 1
**(A)** Immunoblot showing PRL1 levels in HS683 and SW1783 cells transduced with PRL1 or empty vector. **(B)** The quantification of EMT-associated proteins in HS683 and SW1783 cells transfected with PRL1 or empty vector. **(C)** Number of colonies formed by HS683 cells transfected with empty vector or PRL1. ***p* < 0.01; ****p* < 0.001.

Supplementary Figure 2
**(A)** Quantification of protein levels in U87MG and U251 cells transduced with shPRL1#1, shPRL1#2 or shCtrl. **(B)** Number of colonies formed by U87MG cells transfected with shPRL1#1, shPRL1#2 or shCtrl. ***p* < 0.01; ****p* < 0.001.

Supplementary Figure 3
**(A)** Quantification of protein levels in U87MG and U251 cells transduced with shPRL1#1, shPRL1#2 or shCtrl. **(B)** Quantification of PRL1 protein level in U87MG and U251 cells transduced with shPRL1#1 or shCtrl. **(C)** Relative expression of HA-Ub to total His-Snail2. ***p* < 0.01; ****p* < 0.001.

Supplementary Figure 4 Immunoblot showing Snail2 levels in HEK293T cells transfected with siRNAs screened from the DUB siRNA library targeting multiple DUB genes.

Supplementary Figure 5
**(A)** USP5 and USP10 mRNA levels in HEK293T cells with PRL1 knockdown. **(B)** USP5 and USP10 mRNA levels in HEK293T cells overexpressing PRL1. ***p* < 0.01.

Supplementary Figure 6
**(A)** Quantification of PRL1, Snail2 and EMT-related proteins levels in U87MG and U251 cells co-transfected with shPRL1#1/shCtrl or Snail2/control vector. **(B)** Number of colonies formed by U87MG cells transduced with shPRL1#1 and Snail2 or empty vector. **(C, D)** Related to **Figure 3F** and **Figure 7F**. Mice with xenografts of different GBM cells do not differ in their body weights. ***p* < 0.01; ****p* < 0.001; *n.s.*, not significant.

Supplementary Figure 7 Quantification of PRL1 and Snail2 levels in 12 freshly collected clinical GBM tissue samples.

## Abbreviations

ATCC, American Type Culture Collection; NHAs, Normal human astrocytes; GEPIA, Gene Expression Profiling Interactive Analysis; PRL1, Phosphatase of Regenerating Liver 1; EMT, Epithelial–mesenchymal transition; USP36, Ubiquitin specific protease 36; GBM, Glioblastoma multiforme.
