# Supplementary figures and images for: PRL1 Promotes Glioblastoma Invasion and Tumorigenesis via Activating USP36-Mediated Snail2 Deubiquitination
Source: Front Oncol. 2022 Jan 17;11:795633. doi: 10.3389/fonc.2021.795633 (PMC8801937; doi:10.3389/fonc.2021.795633)

A

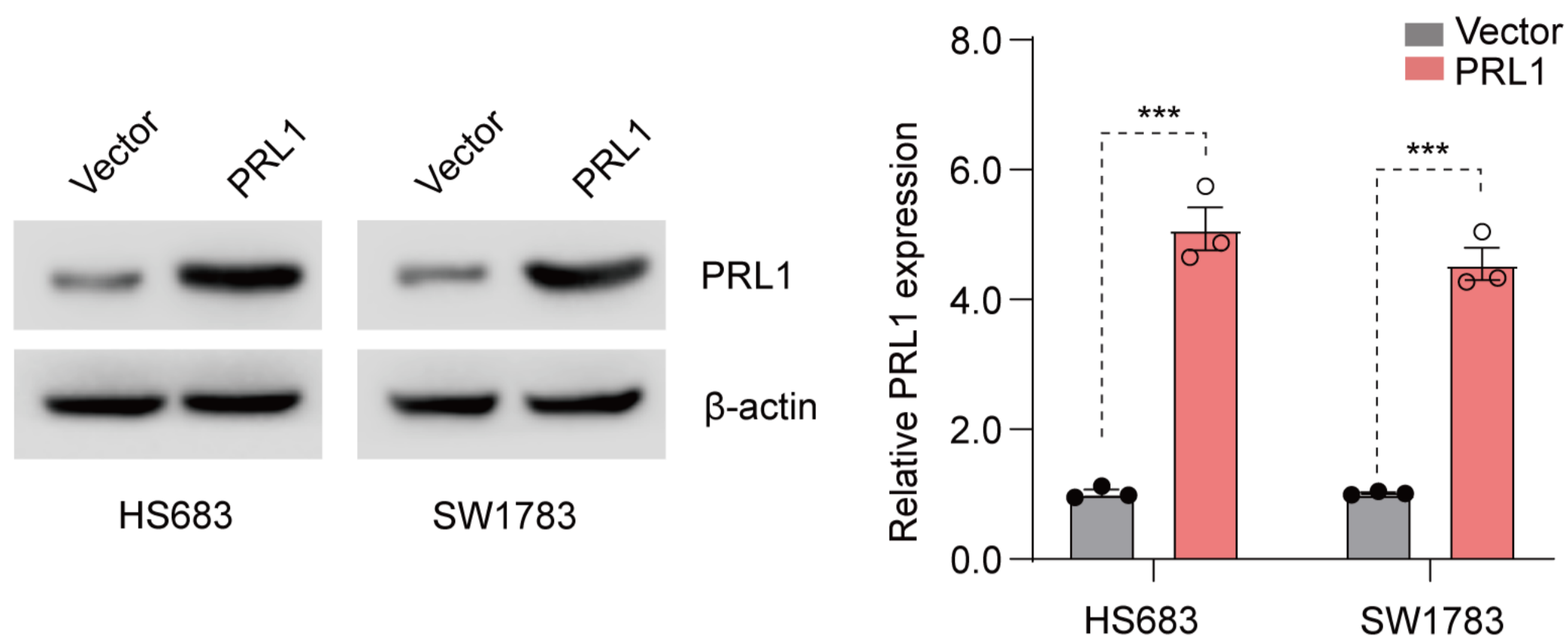

B

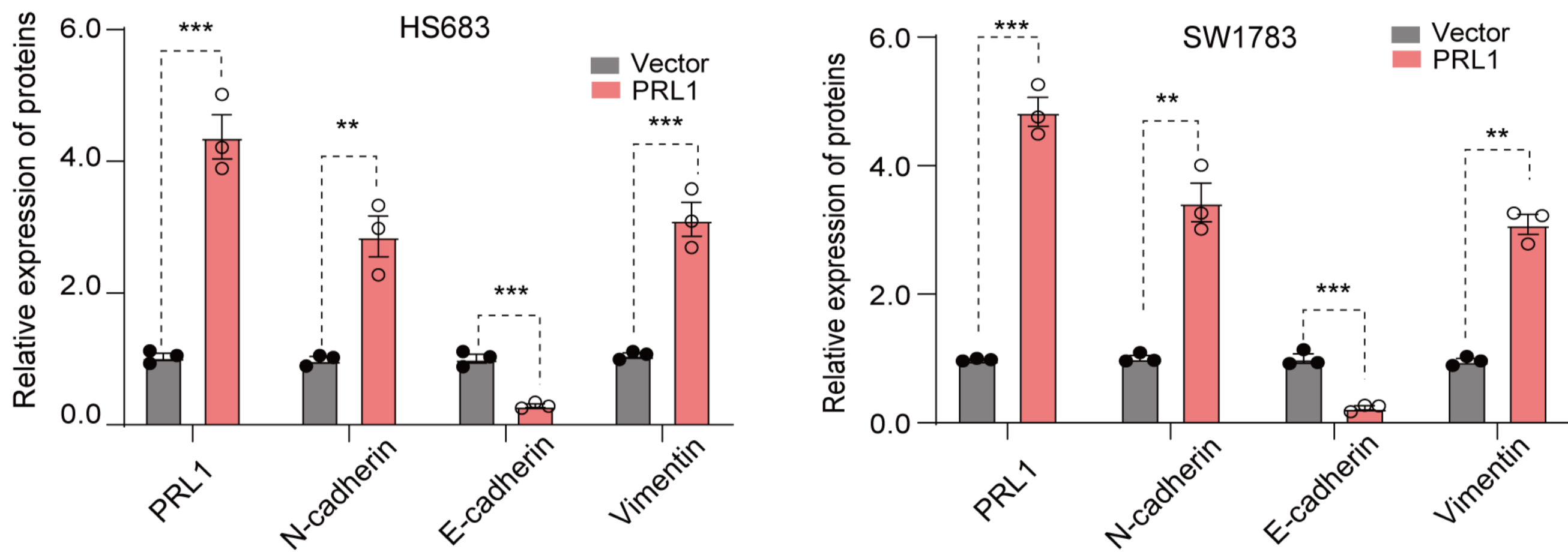

C

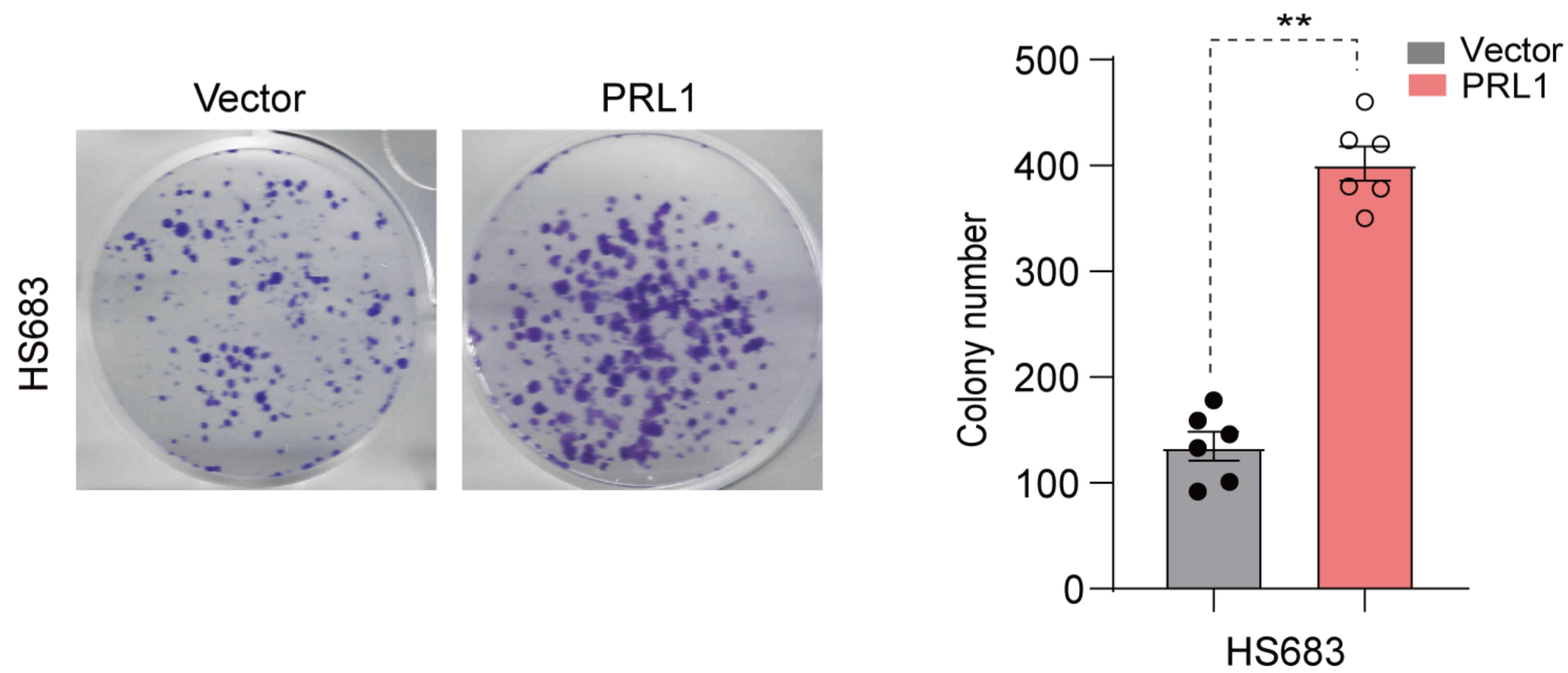

A

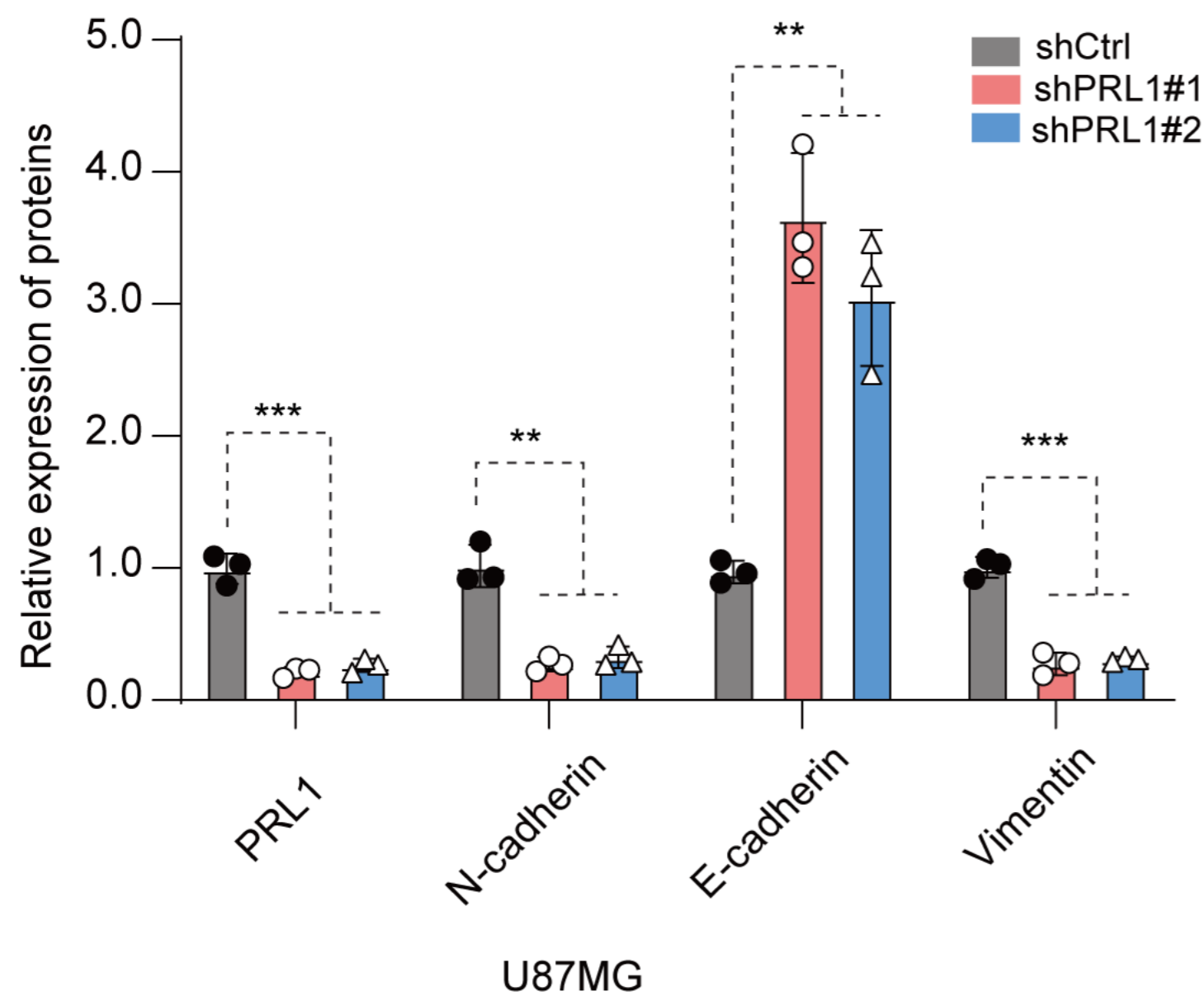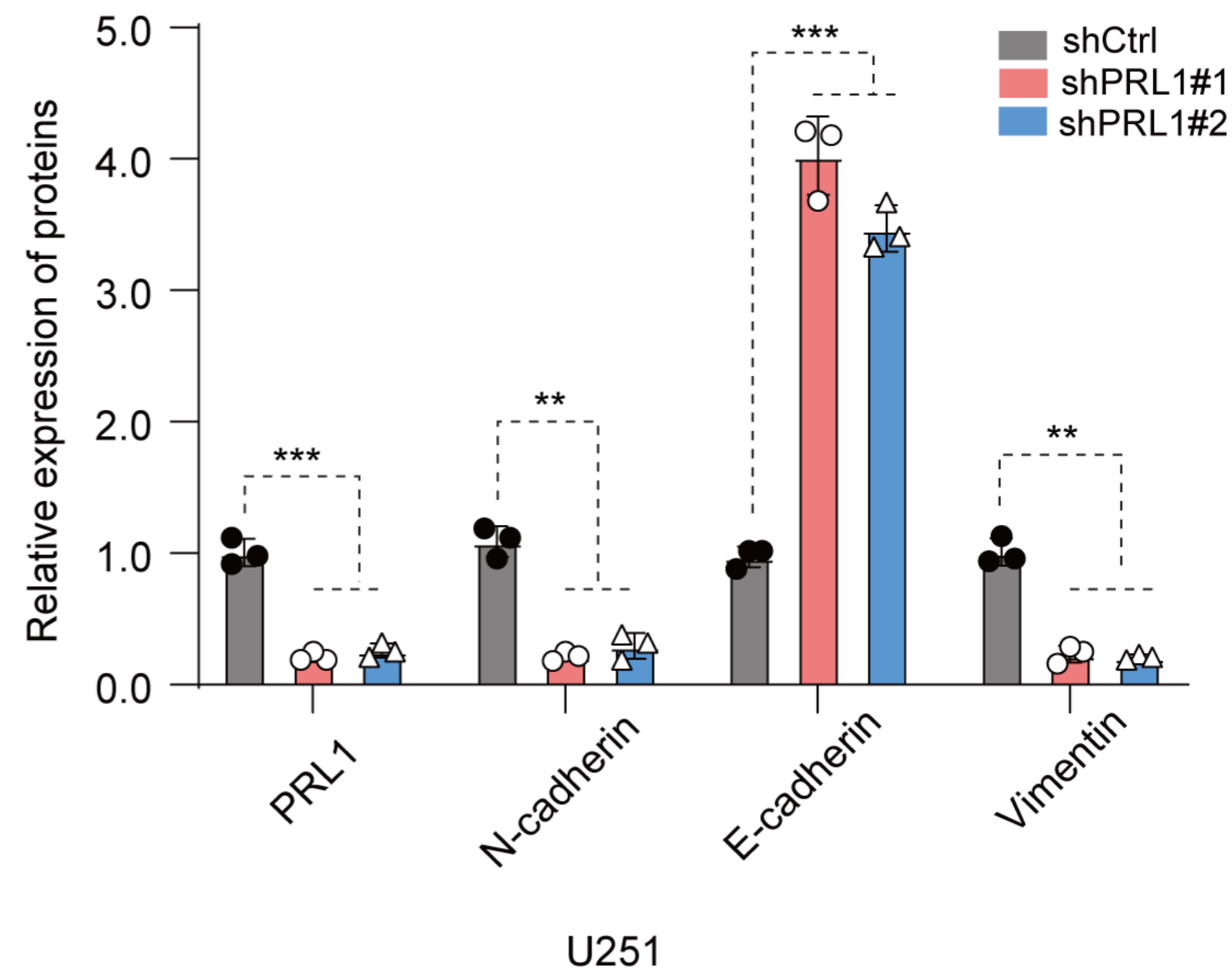

B

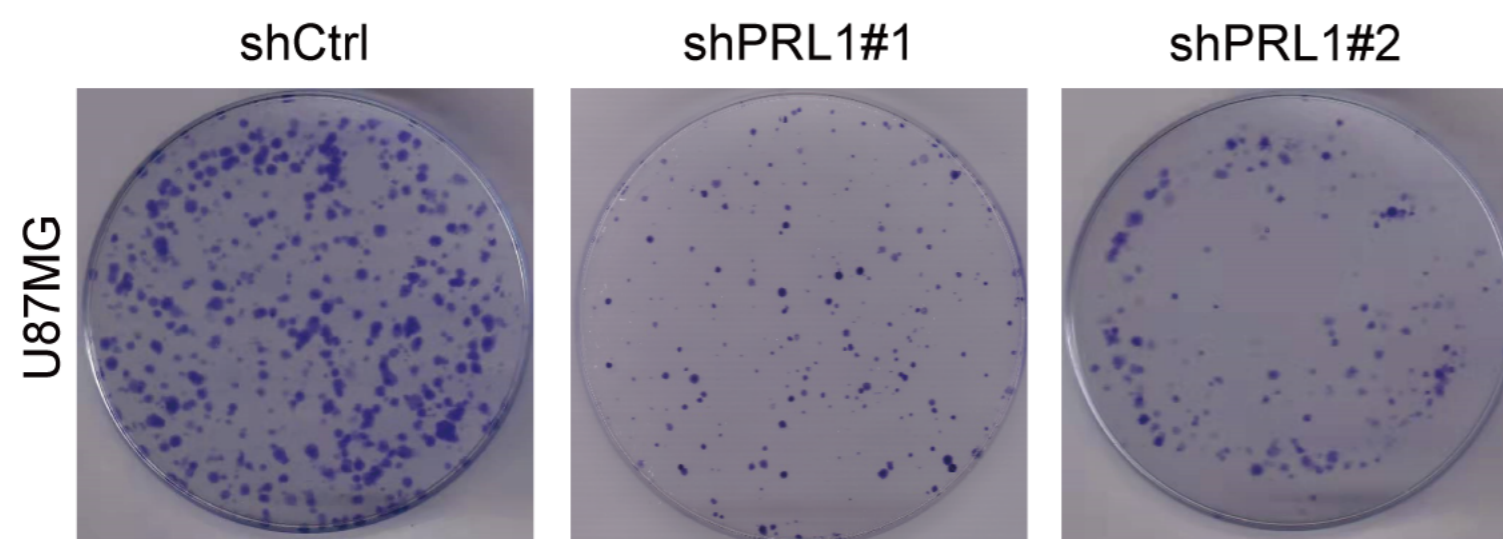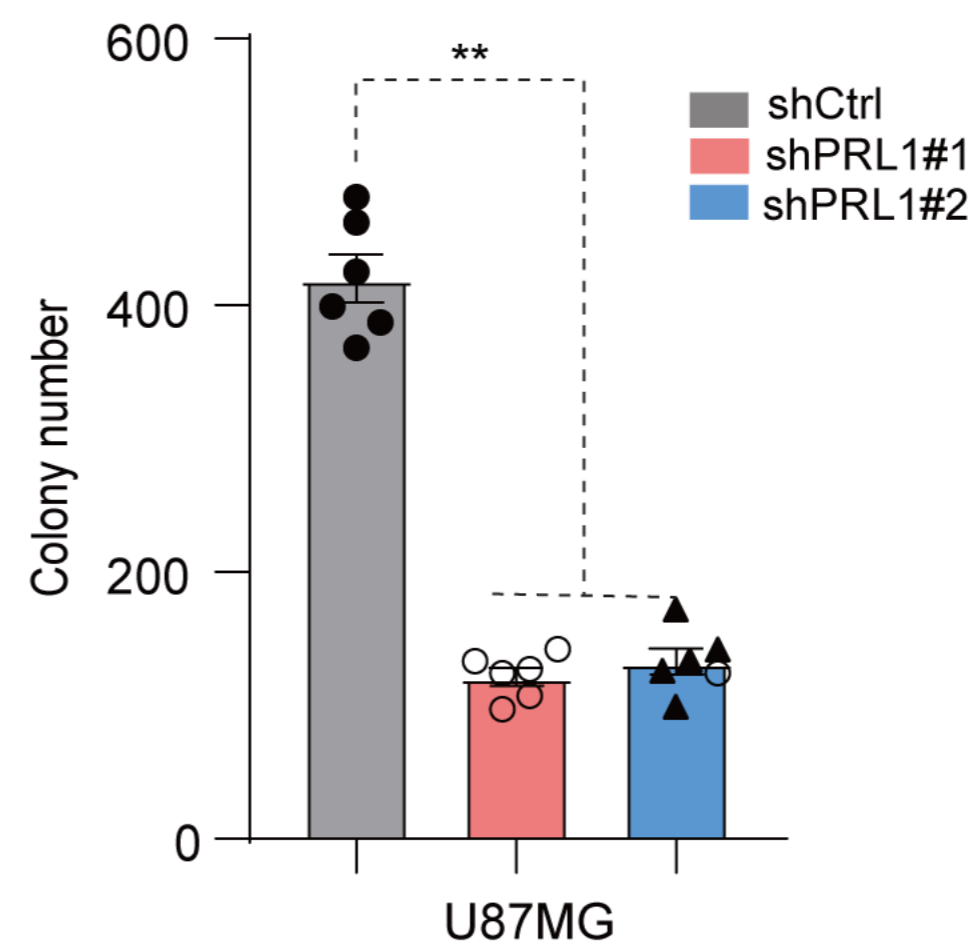

A

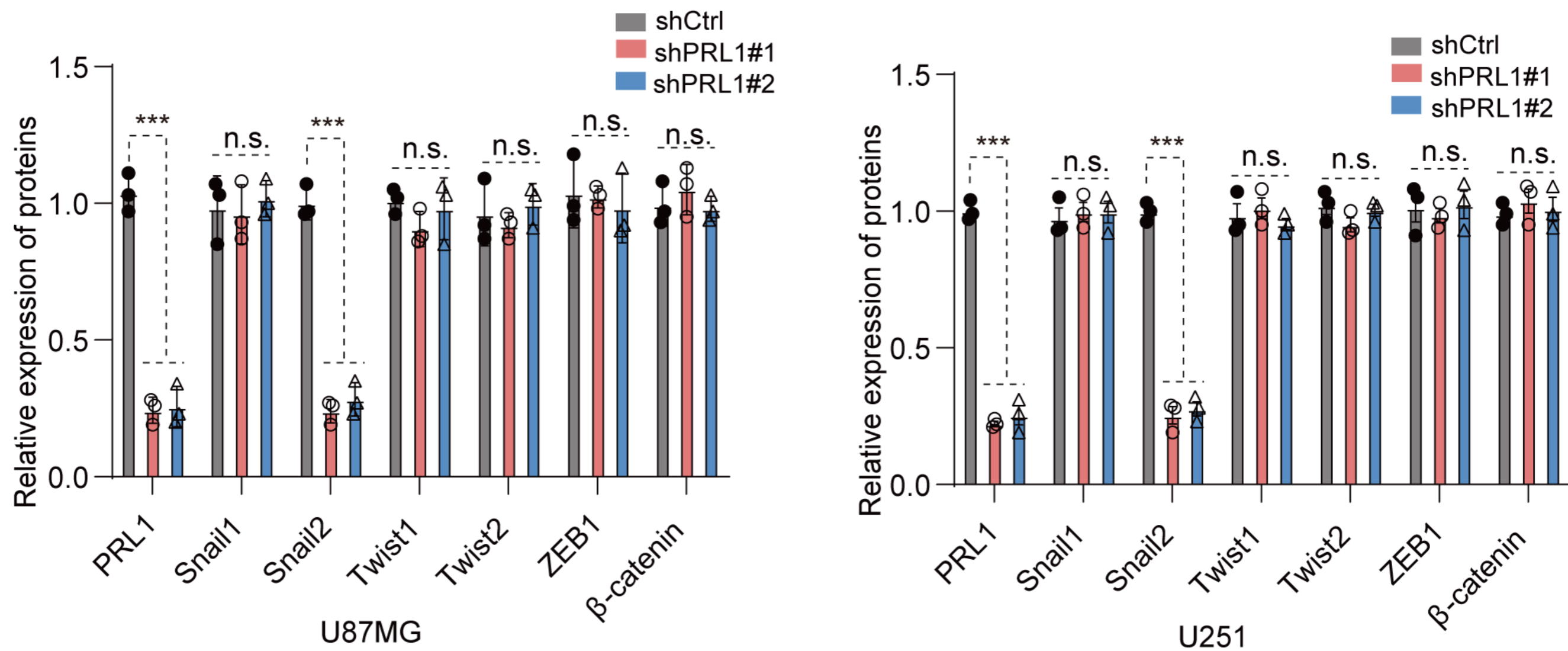

B

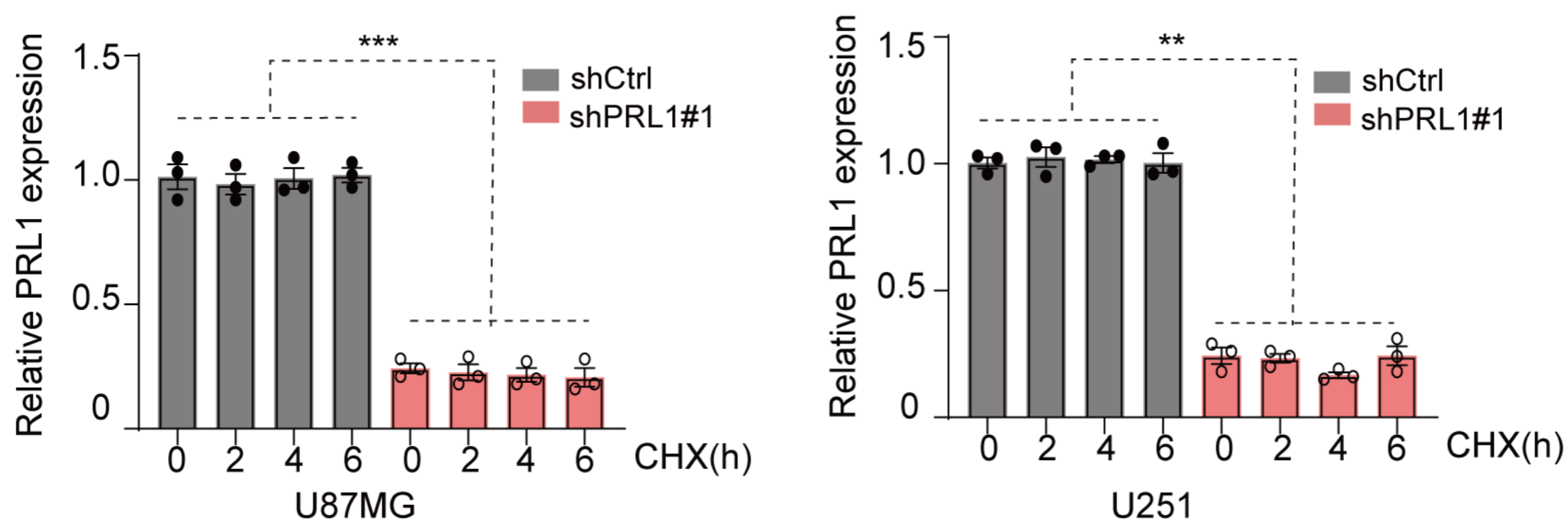

C

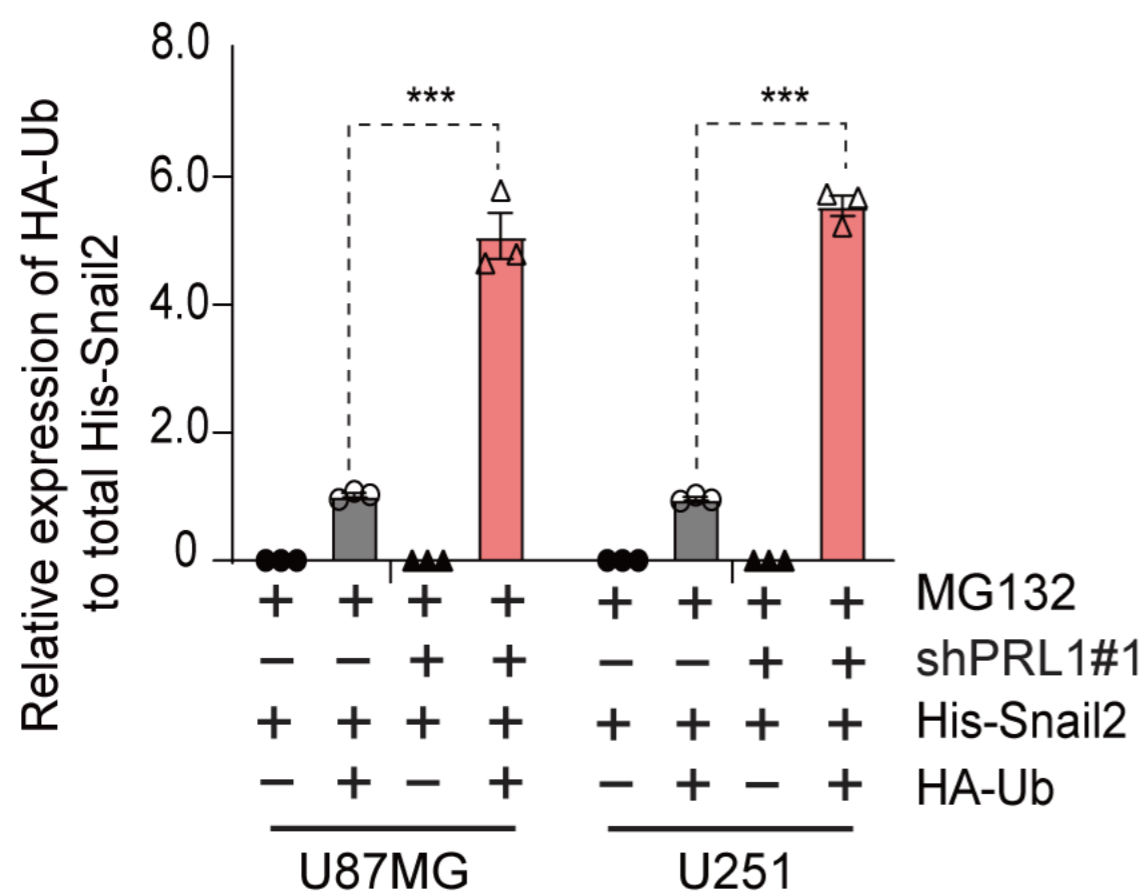

Supplemental Fig. 4

A

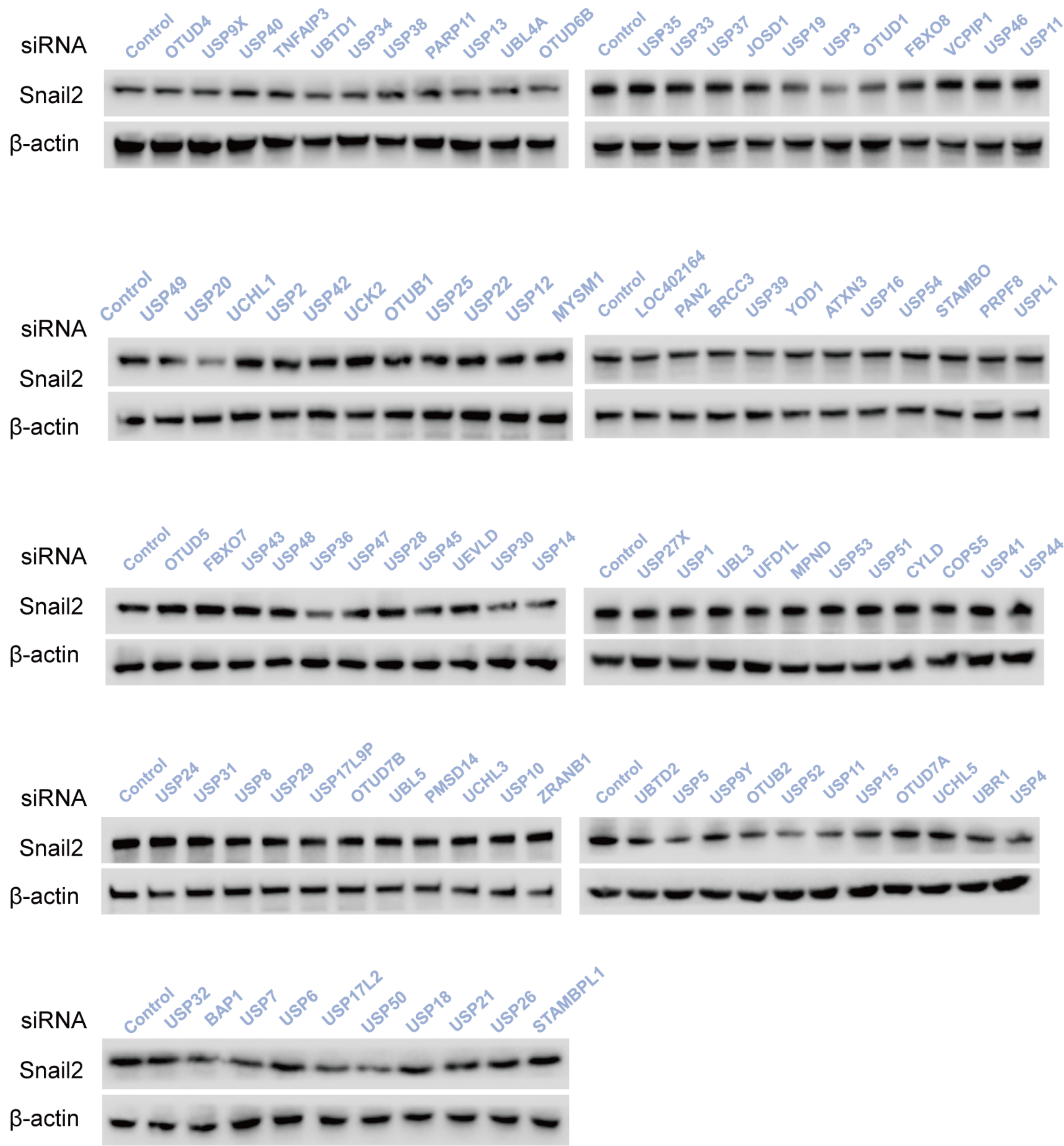

A

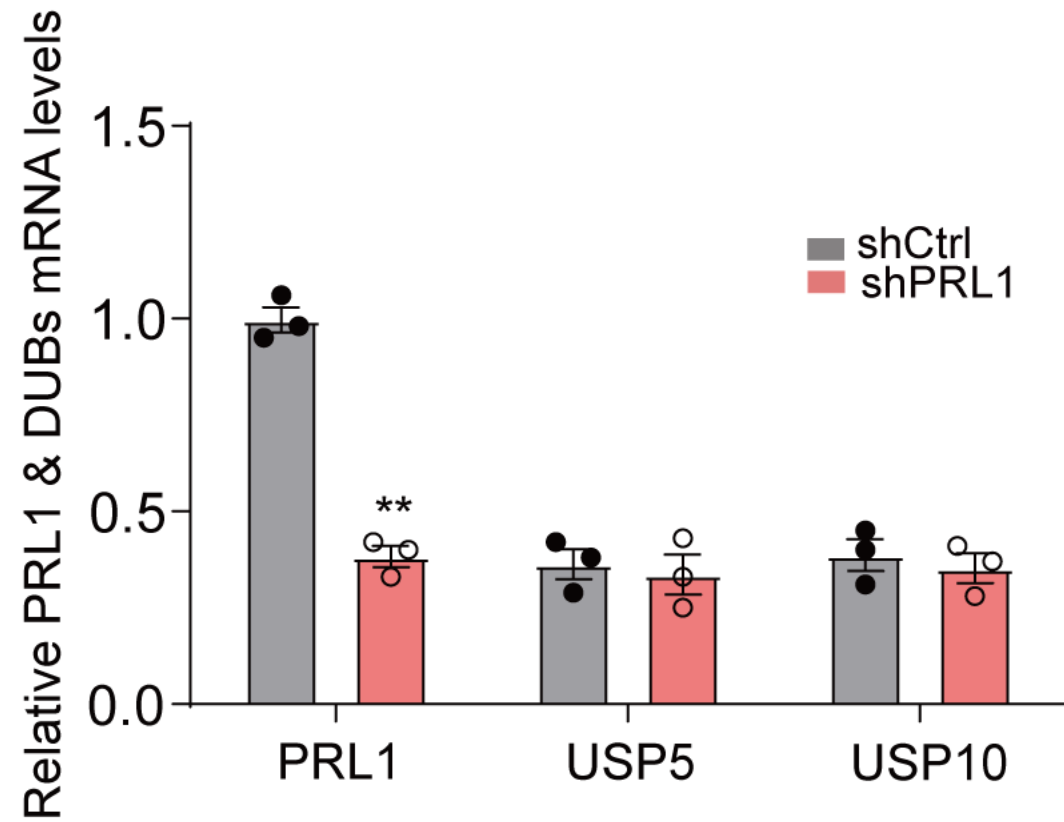

B

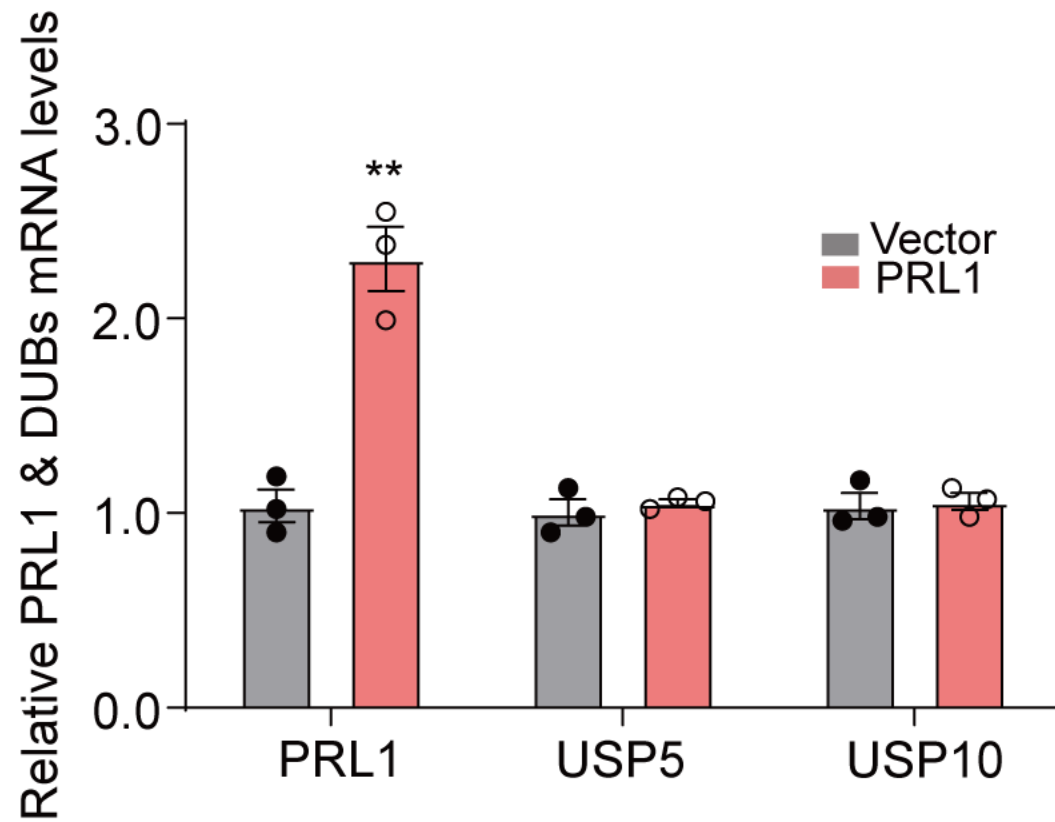

Supplemental Fig. 6

A

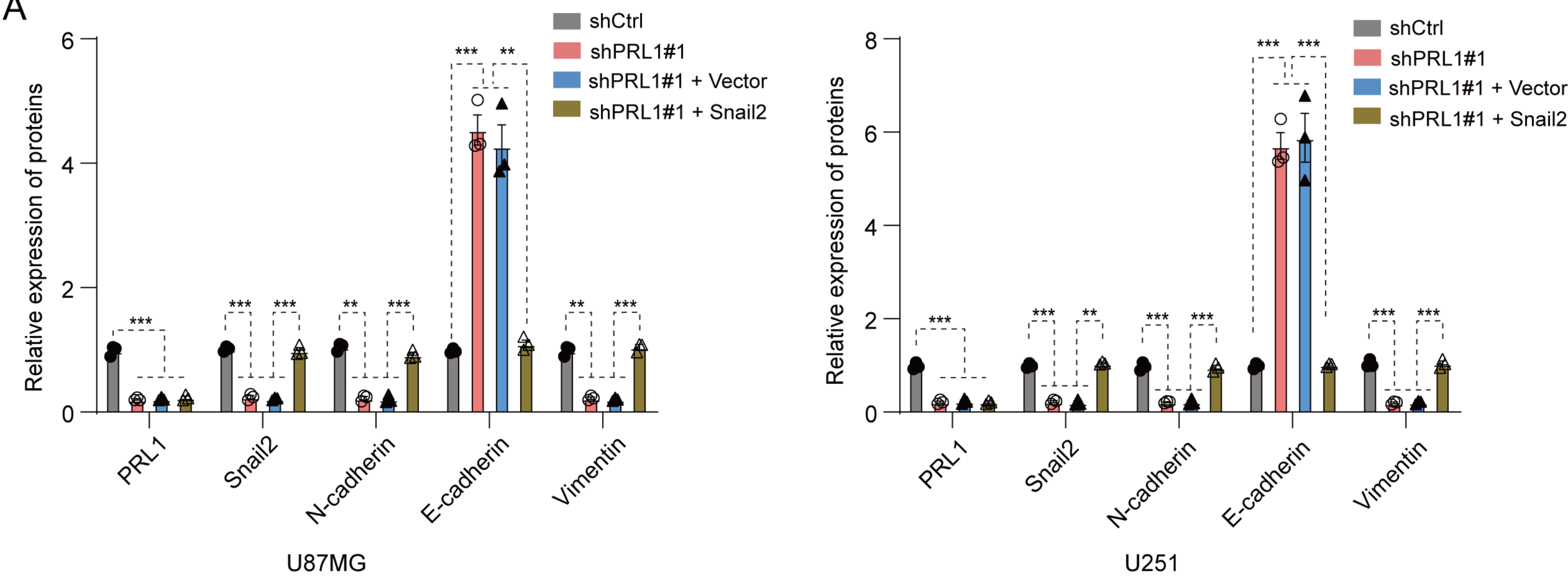

B

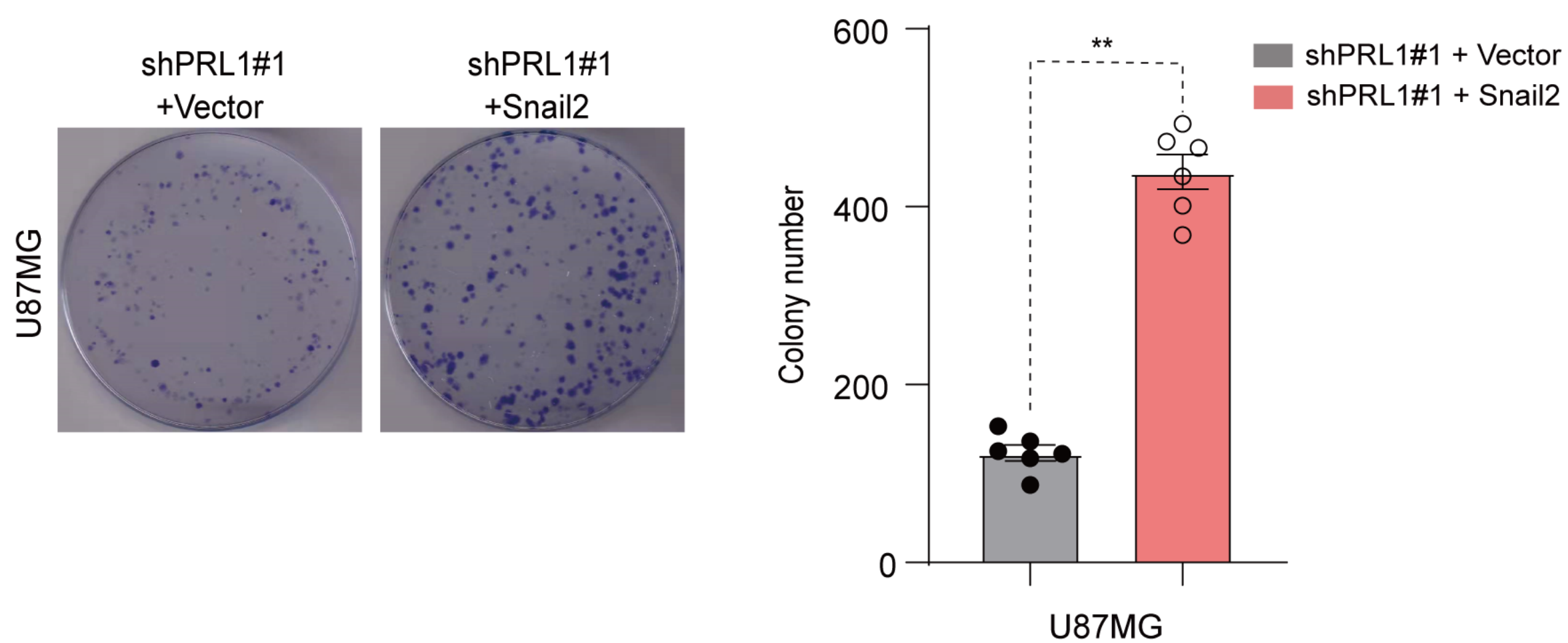

C

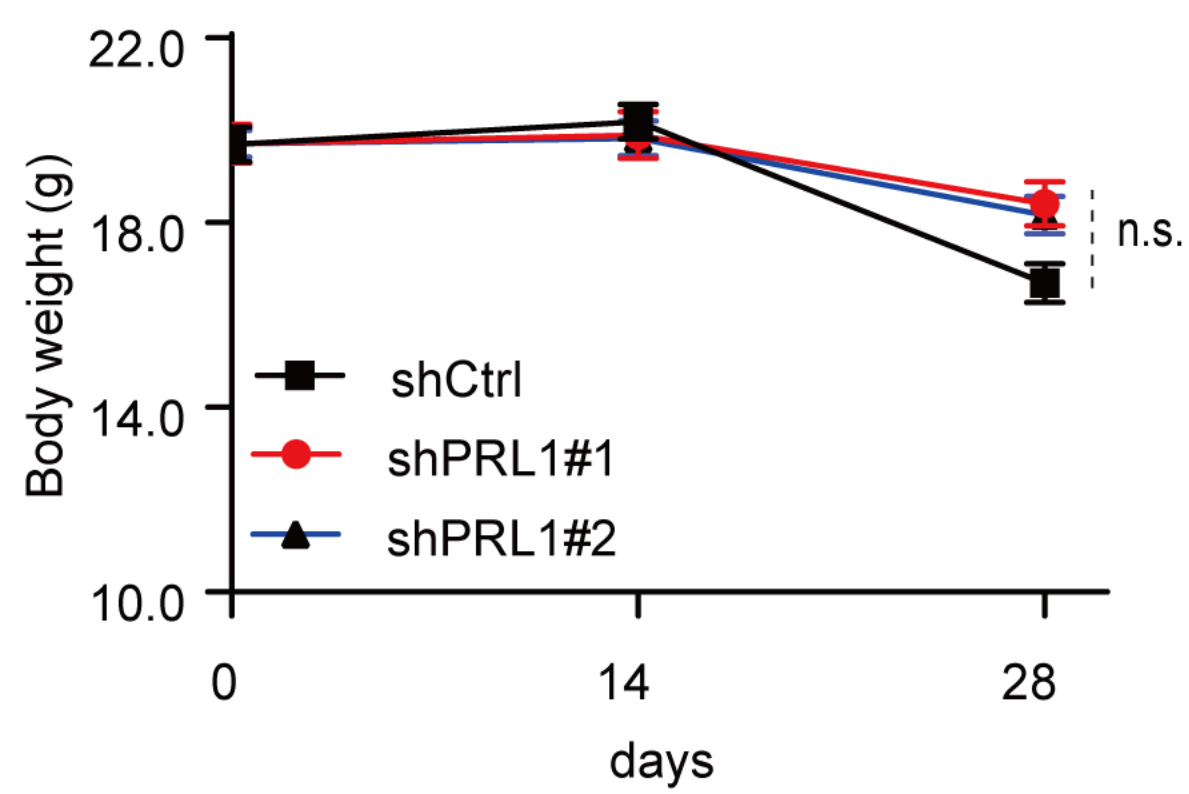

D

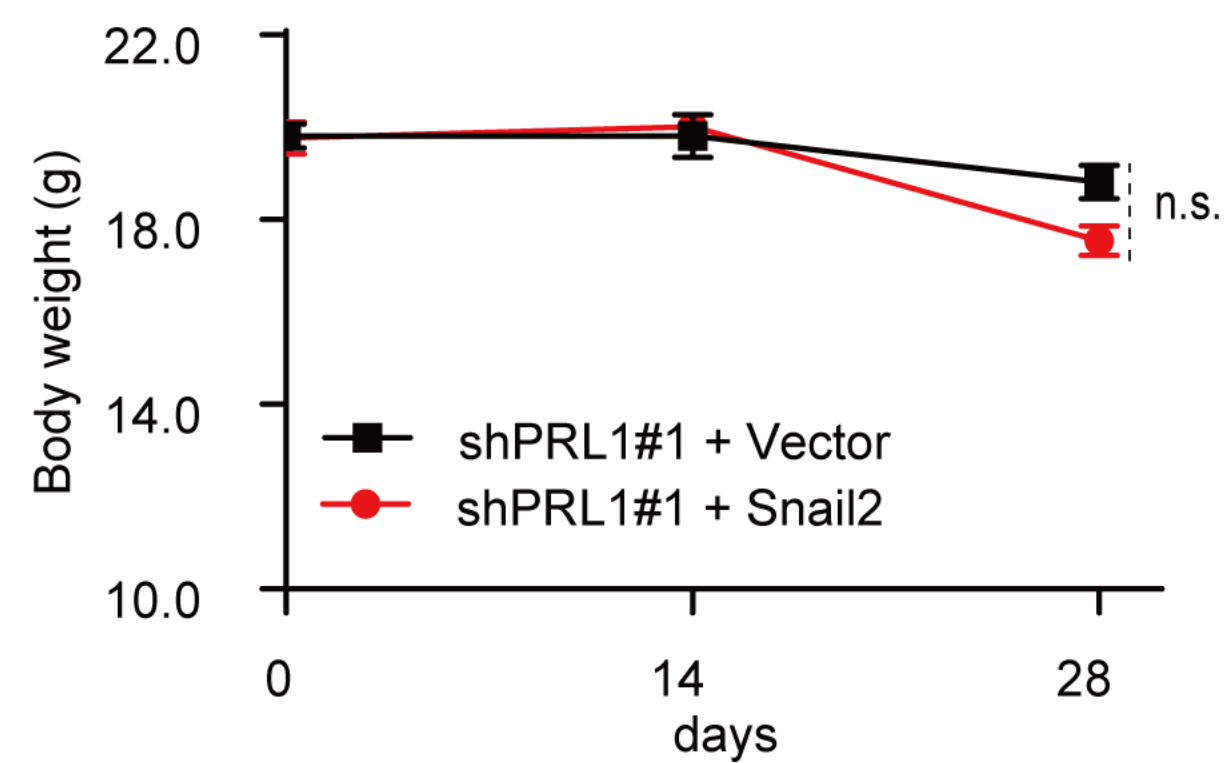

Supplemental Fig. 7

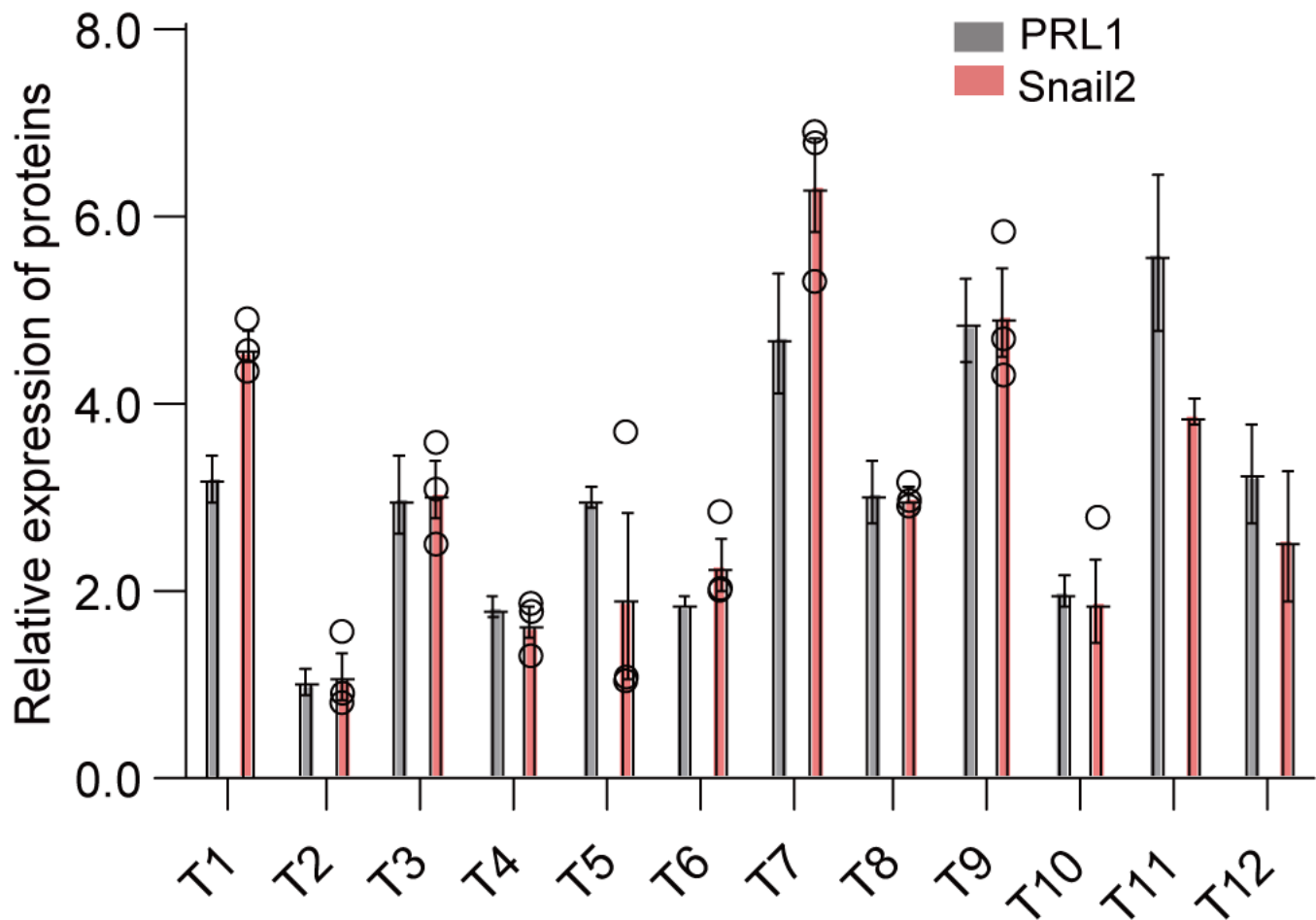

Supplement: Supplementary Figure 1 — (A) Immunoblot showing PRL1 levels in HS683 and SW1783 cells transduced with PRL1 or empty vector. (B) The quantification of EMT-associated proteins in HS683 and SW1783 cells transfected with PRL1 or empty vector. (C) Number of colonies formed by HS683 cells transfected with empty vector or PRL1. **p < 0.01; ***p < 0.001. [file DataSheet_1.pdf]
