# Supplementary material for: PRL1 Promotes Glioblastoma Invasion and Tumorigenesis via Activating USP36-Mediated Snail2 Deubiquitination
Source: Front Oncol. 2022 Jan 17;11:795633. doi: 10.3389/fonc.2021.795633 (PMC8801937; doi:10.3389/fonc.2021.795633)
Supplement: Supplementary file 2 [file Table_1.docx]

**Supplementary Table S1. 26 GBM patients clinicopathological characteristic**


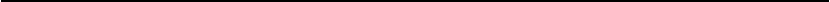


Characteristic Value


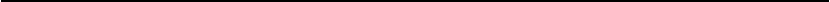


| Total samples (n) | 26 |
| --- | --- |
| Sex (n) |  |
| Male | 12 |
| Female | 14 |
| Medium age, years (range) | 50 (22-73) |
| Tumor location |  |
| Frontal | 15 |
| Non-frontal | 11 |
| Medium KPS (range) | 80 (40-90) |


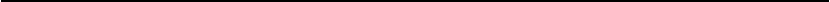


Abbreviations: KPS, Karnofsky performance status; WHO, World Health Organization.

**Supplementary Table S2. 62 glioma patients clinicopathological characteristic**


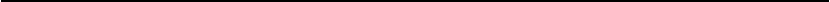


Characteristic Value


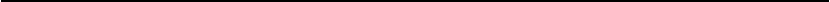


| Total samples (n) | 62 |
| --- | --- |
| Sex (n) |  |
| Male | 33 |
| Female | 29 |
| Medium age, years (range) | 52 (24-77) |
| Tumor location |  |
| Frontal | 35 |
| Non-frontal | 27 |
| Medium KPS (range) | 70 (40-90) |
| WHO grade |  |
| grade I | 8 |
| grade II | 11 |
| grade III | 17 |
| grade IV | 26 |


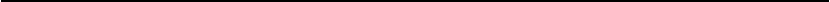


Abbreviations: KPS, Karnofsky performance status; WHO, World Health Organization.
